# GC heterogeneity reveals sequence-structures evolution of angiosperm ITS2

**DOI:** 10.1186/s12870-023-04634-9

**Published:** 2023-12-01

**Authors:** Yubo Liu, Nan Liang, Qing Xian, Wei Zhang

**Affiliations:** 1 Marine College, Shandong University, Weihai, 264209 China; 2 Division of Physical Biology, CAS Key Laboratory of Interfacial Physics and Technology, Shanghai Institute of Applied Physics, Chinese Academy of Sciences, University of Chinese Academy of Sciences, Shanghai, 201800 China; 3 Allergy Department, State Key Laboratory of Complex Severe and Rare Diseases, Peking Union Medical College Hospital, Chinese Academy of Medical Sciences and Peking Union Medical College, Beijing, 100730 China

**Keywords:** ITS2 content, GC-biased gene conversion, Thermodynamic stability, Secondary structure

## Abstract

**Background:**

Despite GC variation constitutes a fundamental element of genome and species diversity, the precise mechanisms driving it remain unclear. The abundant sequence data available for the ITS2, a commonly employed phylogenetic marker in plants, offers an exceptional resource for exploring the GC variation across angiosperms.

**Results:**

A comprehensive selection of 8666 species, comprising 165 genera, 63 families, and 30 orders were used for the analyses. The alignment of ITS2 sequence-structures and partitioning of secondary structures into paired and unpaired regions were performed using 4SALE. Substitution rates and frequencies among GC base-pairs in the paired regions of ITS2 were calculated using RNA-specific models in the PHASE package. The results showed that the distribution of ITS2 GC contents on the angiosperm phylogeny was heterogeneous, but their increase was generally associated with ITS2 sequence homogenization, thereby supporting the occurrence of GC-biased gene conversion (gBGC) during the concerted evolution of ITS2. Additionally, the GC content in the paired regions of the ITS2 secondary structure was significantly higher than that of the unpaired regions, indicating the selection of GC for thermodynamic stability. Furthermore, the RNA substitution models demonstrated that base-pair transformations favored both the elevation and fixation of GC in the paired regions, providing further support for gBGC.

**Conclusions:**

Our findings highlight the significance of secondary structure in GC investigation, which demonstrate that both gBGC and structure-based selection are influential factors driving angiosperm ITS2 GC content.

**Supplementary Information:**

The online version contains supplementary material available at 10.1186/s12870-023-04634-9.

## Background

The Guanine and cytosine (GC) content is crucial in shaping genetic and species diversity due to several factors. Firstly, DNA regions with high GC content are more stable when exposed to extreme temperatures compared to GC-poor regions. This increased stability explains the higher GC content observed in the DNA of thermotolerant microorganisms [1, 2], species capable of thriving in cold and/or dry climates [3], and warm-blooded vertebrates relative to their cold-blooded counterparts [4, 5]. Secondly, genes with high GC content contain more CG dinucleotides, resulting in greater variability in gene expression [6, 7]. Lastly, the synthesis of GC bases requires more biochemical resources compared to AT bases [8], which may explain why plants with large genomes tend to have lower genomic GC content [3, 9, 10]. Numerous studies have shown that GC content contributes to species diversity and adaptation in various organisms. Prokaryotic genomes exhibit a wide range of GC content, ranging from 13 to 75% [11, 12], enabling bacteria to adapt to diverse environments. In certain vertebrate genomes, the distribution of GC-rich and GC-poor regions appears distinct, resulting in an unconventional variation of intra-genomic composition known as isochore [13, 14]. Among plant genomes, grass exhibits the highest and most heterogeneous GC content with a bimodal distribution pattern [10, 15, 16]. Therefore, gaining a deeper understanding of GC variation can provide valuable insights into gene and genome evolution.

There are three hypotheses to explain GC content variation, including selection, mutational bias, and GC-biased gene conversion (gBGC). The selection hypothesis mainly focuses on the elevated GC content of coding sequences, wherein the translation prefers G/C synonymous codons, especially at the third codons [17, 18]. Mutational bias has been discovered in the early stages of DNA replication, where the richer G/C free nucleotides are more likely to mis-incorporate during the polymerization process, resulting in an enriched GC content in the newly synthesized chains [19]. gBGC occurs at the meiotic recombination region, wherein the heterozygous sites in hetero-duplexes create mismatches (A:G, A:C, T:G, and T:C). When these mismatches are repaired by DNA repair systems, the conversion of GC alleles occurs more frequently than for others alleles [20]. This GC-biased gene conversion is therefore expected to bring about an enrichment of GC content in genomic DNA regions with a high recombination rate [21].

Despite the fact that all these hypotheses are plausible in certain evolutionary scenarios, one single hypothesis can hardly interpret the GC variation for all genomic regions, possibly due to the complexity of genome components. Under the mutational bias mechanism, for example, the GC content of a region is highly dependent on its replication time and the availability of free nucleotides in the environment, leaving the question of how GC varies within genes and between generations unanswered [14]. Likewise, the selection mechanism mainly works on the coding regions and hardly explains the GC variation of noncoding genes. Alternatively, the gBGC mechanism can affect both coding and noncoding regions and is considered as a neutral process, because it is insusceptible to the fitness effect on the individuals that carry these regions [22]. However, this interpretation is challenged by the observations of GC-based adaptation. For example, the thermophilic bacteria and the warm-blooded vertebrates always have increased genomic GC content, because GC-rich regions have higher thermal stability than AT-rich regions [4]. Similarly, in monocots, species from seasonally cold and/or dry climatic regions have higher GC contents, implying an adaptation of GC-rich DNA during cell freezing and desiccation [3]. Notably, the recombination rate varies considerably within and among species due to a series of genetic and environmental factors [23, 24]. This flexible feature reduced the correlation between recombination rate and GC content when testing the gBGC hypothesis [22, 25]. In conclusion, the current theoretical hypotheses insufficiently explain the variation in GC content at the genomic level, possibly due to the complexity of genomes.

The Internal Transcribed Spacer 2 (ITS2) serves as an excellent resource to assess GC variation within the local genomic region, rather than the entire genome, for several reasons. Firstly, as the most widely utilized phylogenetic marker, the ITS2 has accumulated an extensive collection of sequences that represent a broad spectrum of evolutionary scenarios. Secondly, the posttranscriptional ITS2 has a recognized secondary structure that exhibits a high level of conservation throughout the eukaryota [26–28]. This structural information could greatly facilitate exploring the correlation between GC content and thermal stability. Moreover, since ITS2 does not encode proteins, it can offer a wealth of nucleotide sites for neutral evolution [29]. Thereby, such sites are expected to reserve most of the GC variation during ITS2 evolution.

It is worth noting that ITS2 exists in multiple copies that are tandemly repeated at different chromosomal sites in plants. Despite these copies occur independently, mutations in all of them lead to homogenization through concerted evolution, which involves unequal crossing over or gene conversion during homologous recombination [29]. The simultaneous occurrence of the ITS2 homogenization process and gBGC in recombination events enables testing the hypothesis that the GC content of ITS2 sequences increases as they become more homogeneous, regardless of variation in recombination rates [23, 24]. We thus alternately used the correlation between the homogeneous degree of ITS2 sequences and their GC content level in a lineage to examine the occurrence of gBGC.

In general, previous studies on GC variation patterns have mainly focused on a select few taxa at the genomic level. However, the complexity of the genome hinders the interpretation of GC variation, rendering it unsuitable for any existing theoretical hypotheses. In our recent study, we discovered that the increase in GC content in the short ITS2 region is influenced by both gBGC and structural stability in a specific angiosperm lineage of *Corydalis*. This implies that analyzing short segments is beneficial for investigating GC content [30]. Given the vast number of ITS2 sequences available for primary plant lineages (8666 species and 63 families), the objective of this study is to examine the patterns of GC variation and the underlying mechanisms among angiosperms. The secondary structure of ITS2 was constructed, and a comparison of GC content was made between the stem and loop regions to investigate if there is an association between GC content and thermal stability. Additionally, we used ITS2 nucleotide substitution models to infer the conversion of GC content, under the fundamental assumption that the substitution process is constant within a given lineage [31, 32]. To verify the gBGC hypothesis regarding ITS2 GC variation, we compared base-pair transformations between GC and AU within GC-rich regions.

## Results

### Variability of ITS2 GC content among angiosperms

**Fig. 1:**
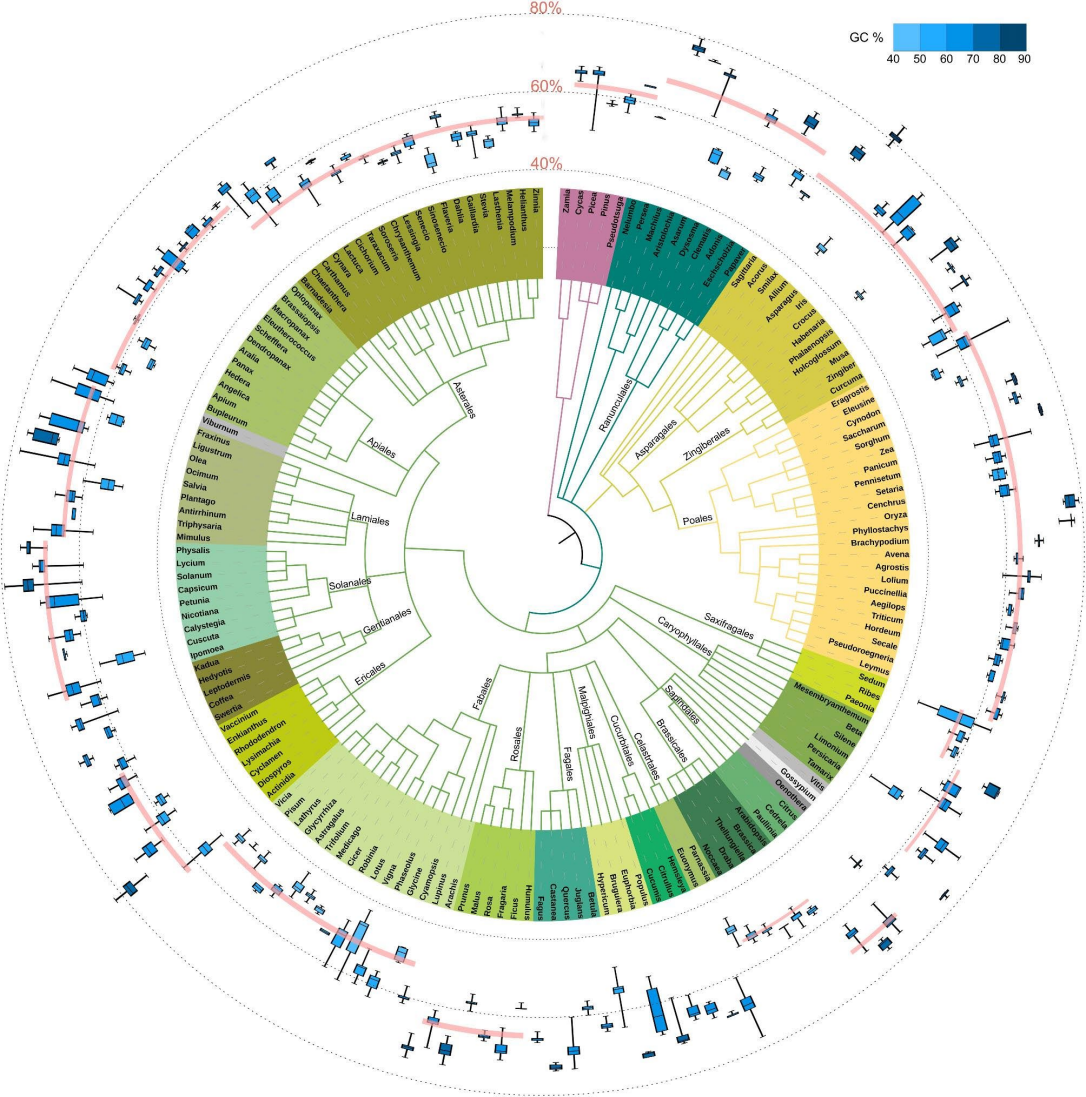
Variation of ITS2 GC content across the 165 angiosperm phylogeny. The three dotted-line circles outside the circular tree represent the GC content intervals, in which the GC boxplots are added behind their generic names. The phylogeny was constructed by using the NCBI taxonomy, which is based on the APG system. The main orders are highlighted with color blocks. The mean ITS2 GC content of each order is indicated by a pink line, and the width of the line represents the 95% confidence interval

**Fig. 2:**
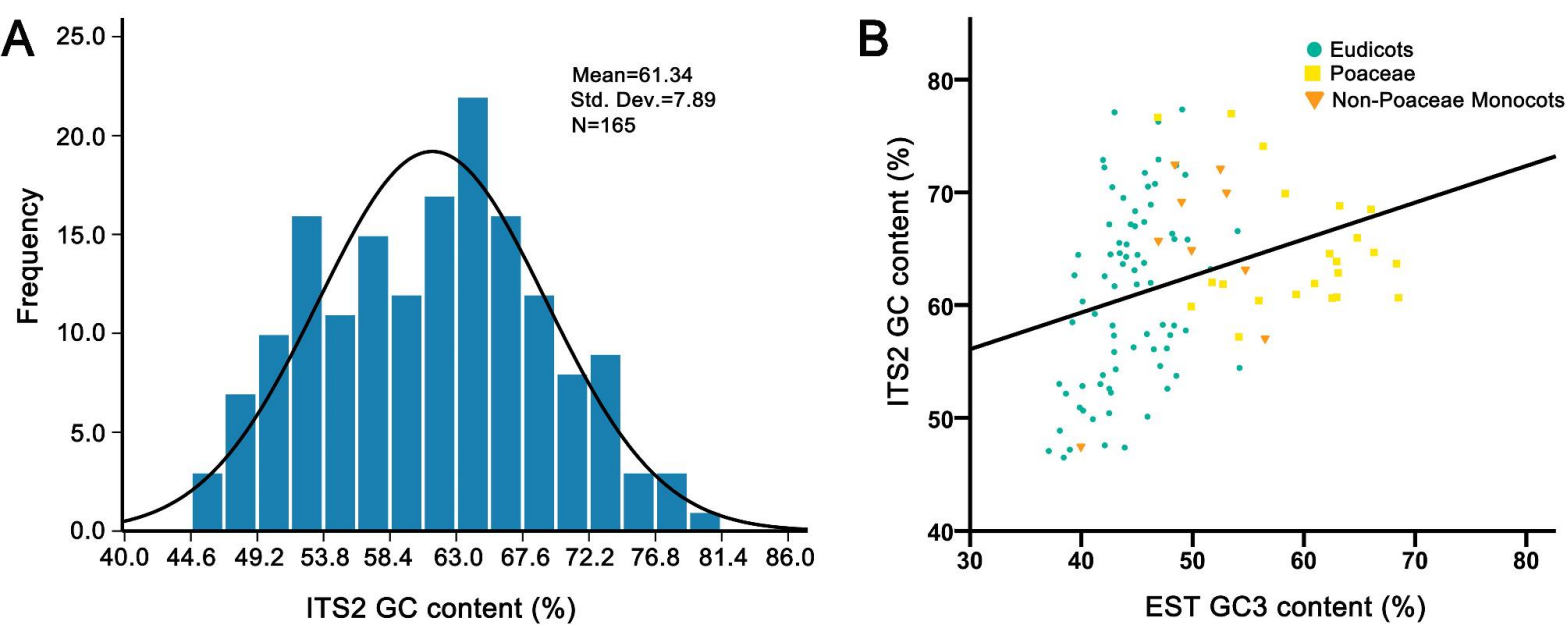
Distribution and variation of ITS2 GC contents among angiosperms. **A** Histogram of ITS2 GC contents across 165 sampled genera showing a normal distribution. **B** a scatter plot illustrating the correlation between ITS2 GC contents and the GC contents at the third codon position of the expressed sequence tags (EST-GC3) among 107 representative genera of Eudicots, Poaceae and the non-Poaceae monocots. Each data point represents the average GC value of a genus

The GC contents of the ITS2 sequence varied considerably from 46.24% (*Flaveria*, Asteraceae) to 81.39% (*Smilax*, Smilacaceae), averaging 61.34% among the 165 genera in the major lineages of angiosperms (Additional file 1: Table S1). They even varied greatly among the closely related species, e.g., the GC content of *Salvia* species varied greatly from 58.26 to 77.83%. Interestingly, we found that GC-rich genera were always more heterogeneous than GC-poor genera; for example, the GC content of *Solanum* was 73.03 ± 2.37% vs. 47.1 ± 0.73% in *Medicago*. In order to examine the distribution of GC content in the ITS2 region among angiosperms, we displayed the GC content of all analyzed genera using an updated classification by the Angiosperm Phylogeny Group (APG III; Fig. 1). Despite their great variation among distinct plant lineages, the frequency distribution of these GC contents was normally distributed among all angiosperms (P = 0.777, Kolmogorov-Smirnov test; Fig. 2A). We divided the angiosperms into eudicot plants, Poaceae plants, and non-Poaceae monocots, given that their genomic GC content has been reported to be distinct from each other. We found that the ITS2 GC contents of the monocots were higher than those of dicots (64.25% vs. 60.53%; Fig. 2B). Within the monocots, the Poaceae and the non-Poaceae plants were almost identical (64.29%±5.85% vs. 64.17%±9.78%), but the Poaceae plants were more homogeneous than those of the non-Poaceae plants (SD: 5.85% vs. 9.78%; Fig. 2). Within the dicots, the ITS2 GC contents of Brassicales (54.21 ± 1.63%), the Fabales (57.54 ± 9.38%) and the Asterales (54.40 ± 5.34%) were obviously lower than other plant orders. The highest ITS2 GC concentration was found in the Solanales (66.09 ± 6.60%) and its most closely related orders. Following Serres-Giardi et al. [15], we statistically analyzed the genera with both the ITS2 and the expressed sequence tags (EST)-GC3 datasets and found that their values were positively corrected (rho = 0.386, *P*<10^− 4^, Spearman correlation; Fig. 2B), indicating that the local ITS2 GC content might be a potential proxy for genomic GC content.

### Heterogeneity of GC content between ITS2 paired and unpaired regions

**Fig. 3:**
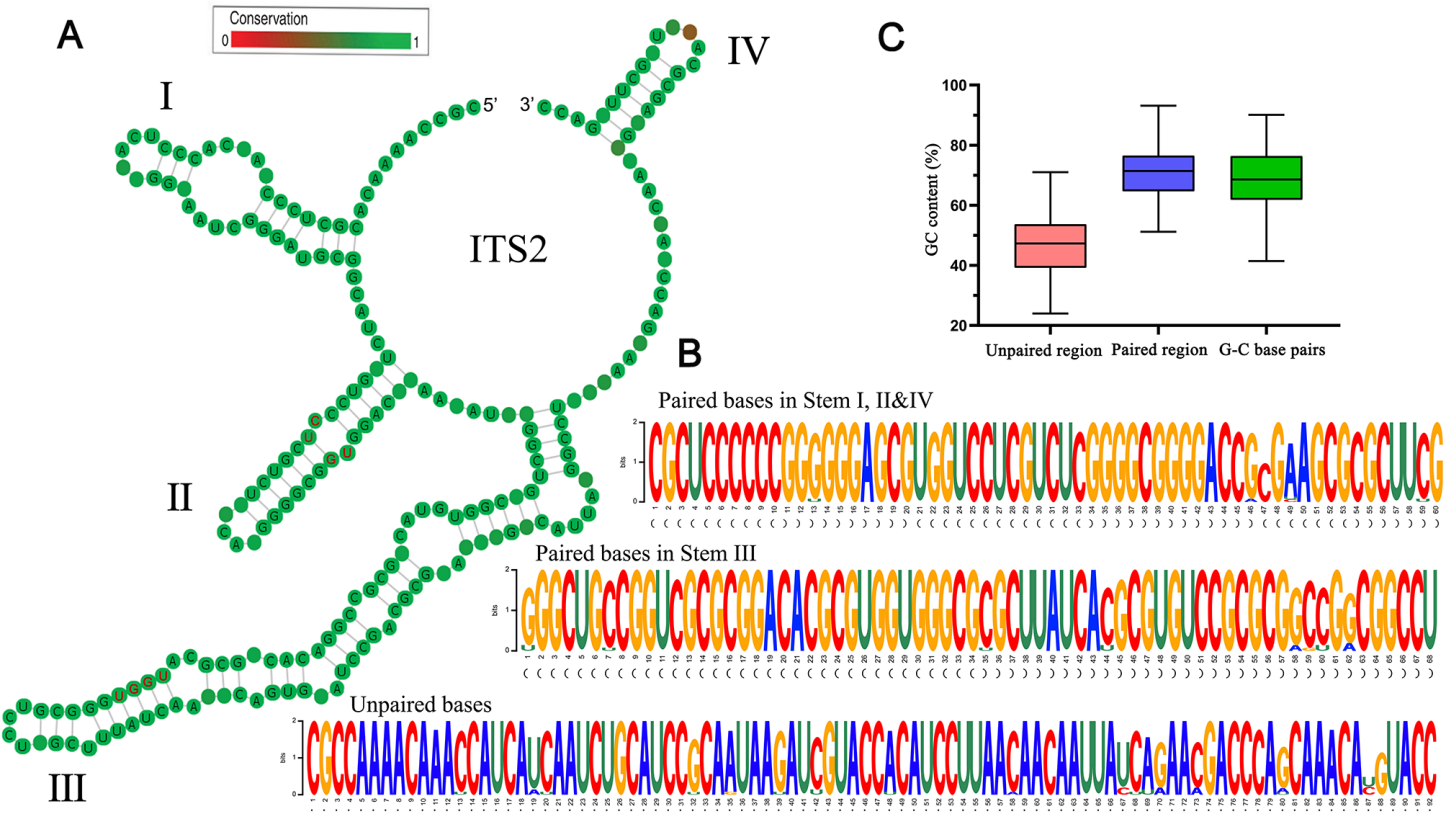
GC distribution in ITS2 secondary structure. **A** An example of the ITS2 consensus secondary structure from genus *Aegilops* (family Poaceae). The four stems are labelled I–IV. The characteristic bulge in stem II, and the UGGU in stem III that are common to nearly all angiosperms, are indicated in red. Degree of conservation over the entire sequences is displayed using color grades ranging from green (conservative) to red (variable). **B** ITS2 sequence logo of the genus *Aegilops* is used to visualize the base composition in different sequence-structure partitions. The overall height of the letter stack in each position indicates the sequence conservation (measured in bits), while the height of letter within the stack represents the relative frequency of the bases at that position. **C** The statistics of GC contents and G-C base pair frequency in ITS2 sequence-structure partitions among the 165 investigated genera

All ITS2 sequences among our 165 investigated genera were folded into a common ‘four-fingered hand’ form of secondary structure, as has been reported before, ensuring the reliability of the following structure-based analyses. For example, helix III was the longest stem, and helix IV was the most variable. In addition, some conserved motifs were also observed, such as the pyrimidine-pyrimidine bulge and a non-canonical U-G base pair in helix II and UGGU in helix III (Fig. 3A). The length of ITS2 ranged from 185 to 324 bp, with an average of 143.16 bp in paired regions and 94.56 bp in unpaired regions. Intriguingly, we found that the GC content in paired regions (GC_p_) was always higher than that in unpaired regions (GC_up_) for each genus in our study (Fig. 3B, C; Additional file 1: Table S1). Taken across all 165 genera, the average GC_p_ was 150.26% of the GC_up_. There are a total of 16 possible base-pair combinations in paired regions, among which the G-C base pair (hereafter the 5’G-C/5’C-G in secondary structure is collectively termed G-C; other combinations are also termed likewise) is particularly important because of their three hydrogen bonds. We found that the G-C base pair always predominated in each ITS2 secondary structure, accounting for 70.69 ± 8.36% of all base pairs in the 165 investigated matrices (Fig. 3C). Taken together, these distinct GC compositions between paired and unpaired regions of ITS2 secondary structure observed here indicate different GC elevating mechanisms have taken place on the ITS2 sequence and emphasize the importance of structural information when analyzing GC content.

### Comparison between equilibrium GC and current GC content

**Fig. 4:**
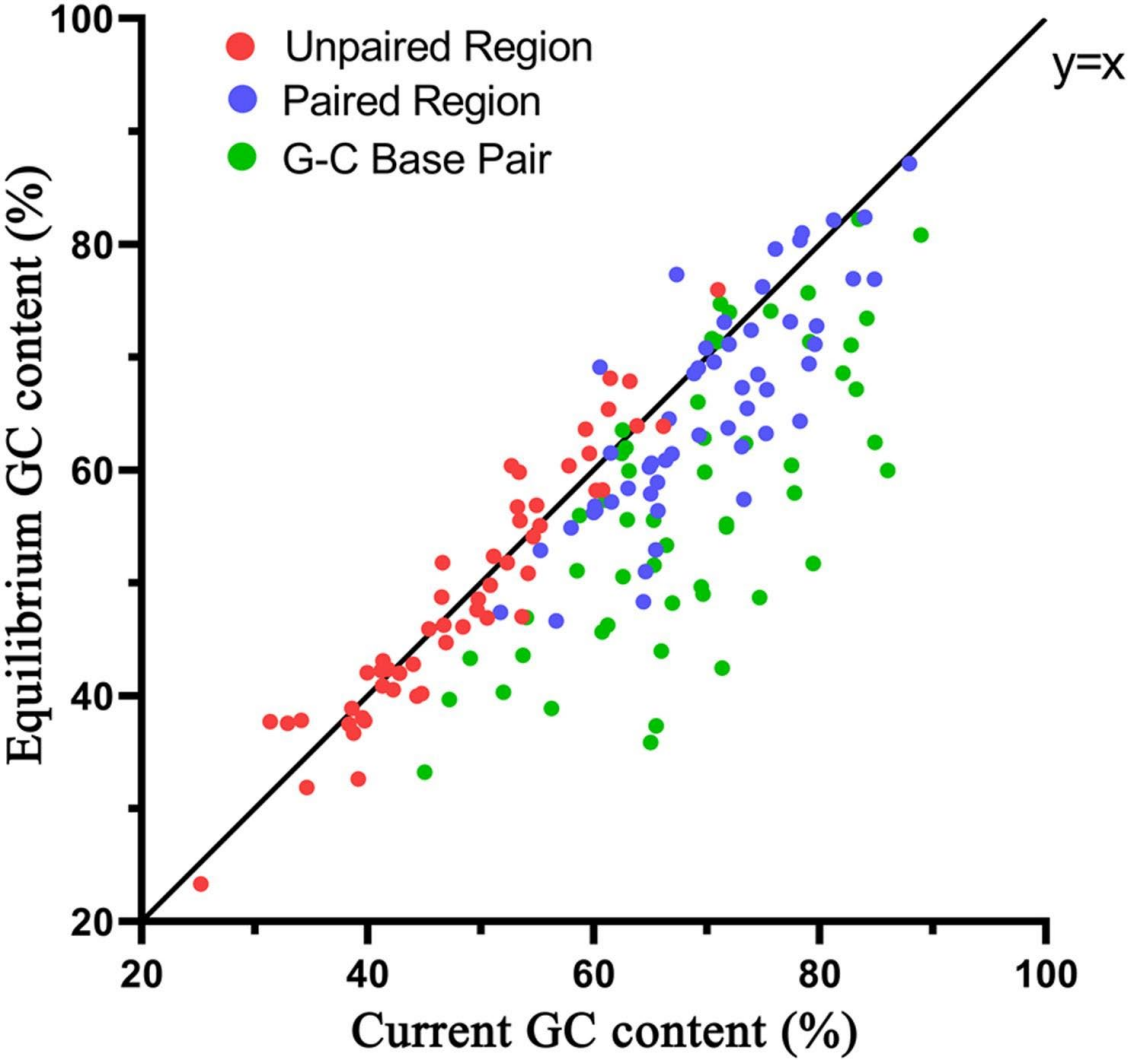
Correlations between the current and the equilibrium GC content (frequency) among 165 ITS2 sequence structures. Each data point represents the average GC value or G-C frequency of a genus

Equilibrium GC content (hereafter termed GC*) refers to the future GC content when sequences evolve convergently at the stationary state, based on the assumption that the patterns of substitution remain constant over time. GC* thus provides clues to infer the evolutionary trend of GC content. We performed Pearson correlation coefficients and observed that, in general, the GC* contents were positively correlated with the current GC content in both the unpaired (*r* = 0.954, *P*<10^− 27^) and paired regions (*r* = 0.827, *P*<10^− 13^). However, there were some different situations between them (Fig. 4; Additional file 1: Table S1). In the unpaired region (GC-poor region), GC* contents were quite similar to current GC contents (P = 0.472), indicating that the equilibrium of the base composition has been reached. By contrast, in the paired region (the GC-rich region), GC* contents were obviously lower than the current GC contents (Fig. 4), suggesting that the equilibrium of base composition has not been reached. Similarly, the current G-C content (frequency) was also positively correlated with the equilibrium G-C content (*r* = 0.710, *P*<10^− 8^). However, the slope of their best-fit regression line was the lowest, indicating that the G-C content is far from equilibrium compared with GC in paired or unpaired regions. When these findings and their structural context are considered together, it appears that the more a GC is involved in the formation of ITS2 secondary structure, the further it is from equilibrium. These observations suggested that the ITS2 structure has been involved in maintaining the current higher GC content.

### Correlation between GC content and nucleotide polymorphism

**Fig. 5:**
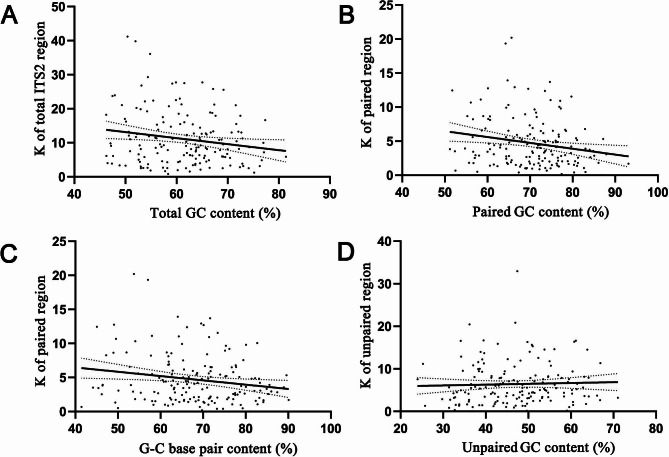
Correlations between the average number of nucleotide differences (K) and GC content (G-C frequency) among 165 ITS2 sequence-structure matrices. Each data point represents the average GC value or G-C frequency for a single genus. The regression line was calculated using Pearson’s correlation, with the error bands represent 95% confidence intervals based on a binomial model. **A** Comparison of the GC content and the K value for the entire ITS2 sequence. **B** Comparison of the GC content and the K value for the ITS2 paired regions. **C** Comparison of the G-C frequency and the K value for the ITS2 paired regions. **D** Comparison of the GC content and the K value for the ITS2 unpaired regions

ITS2 is a well-known tandemly repeated gene region with hundreds to thousands of copies at one or more chromosomal locations. Eventually, these different paralogous copies become homogenized after concerted evolution via recombination. Under the gBGC model, recombination could also elevate GC content. We thus expected that if gBGC worked on ITS2, then the elevated GC should be accompanied by sequence homogenization, leading to a decrease in the average number of nucleotide differences (K). For example, the K was 18.24 in the GC-lowest genus *Flaveria* (GC = 46.24%), compared to that of 5.90 in the GC-highest genus *Smilax* (GC = 81.39%). We calculated the K value and the contents of GC, GC_p_, GC_up_^,^ and G-C in each genus and found the same trend in most cases: the GC increased as K decreased (Additional file 1: Table S1). We performed Pearson correlation coefficients between K and GC, G-C, GC_p_^,^ and GC_up_ respectively, as shown in Fig. 5. Overall, the K value was more or less negatively correlated with GC (*r* = -0.179, *P* = 0.022), GC_p_ (*r* = -0.201, *P* = 0.009) and G-C content (*r* = -0.186, *P* = 0.017), supporting that the elevated GC occurred mainly with sequence homogenization (Fig. 5A-C). However, some other factors could also affect GC content since a high GC content was not necessary with a low K value in a certain number of ITS2 matrices. Notably, we found that the K value was almost irrelevant with the increased GC_up_ contents (*r* = 0.038, *P* = 0.628). Taken together, there were probably not merely gBGCs involved in ITS2 GC enrichment.

### Base-pair mutational dynamics in ITS2 secondary structure

**Fig. 6:**
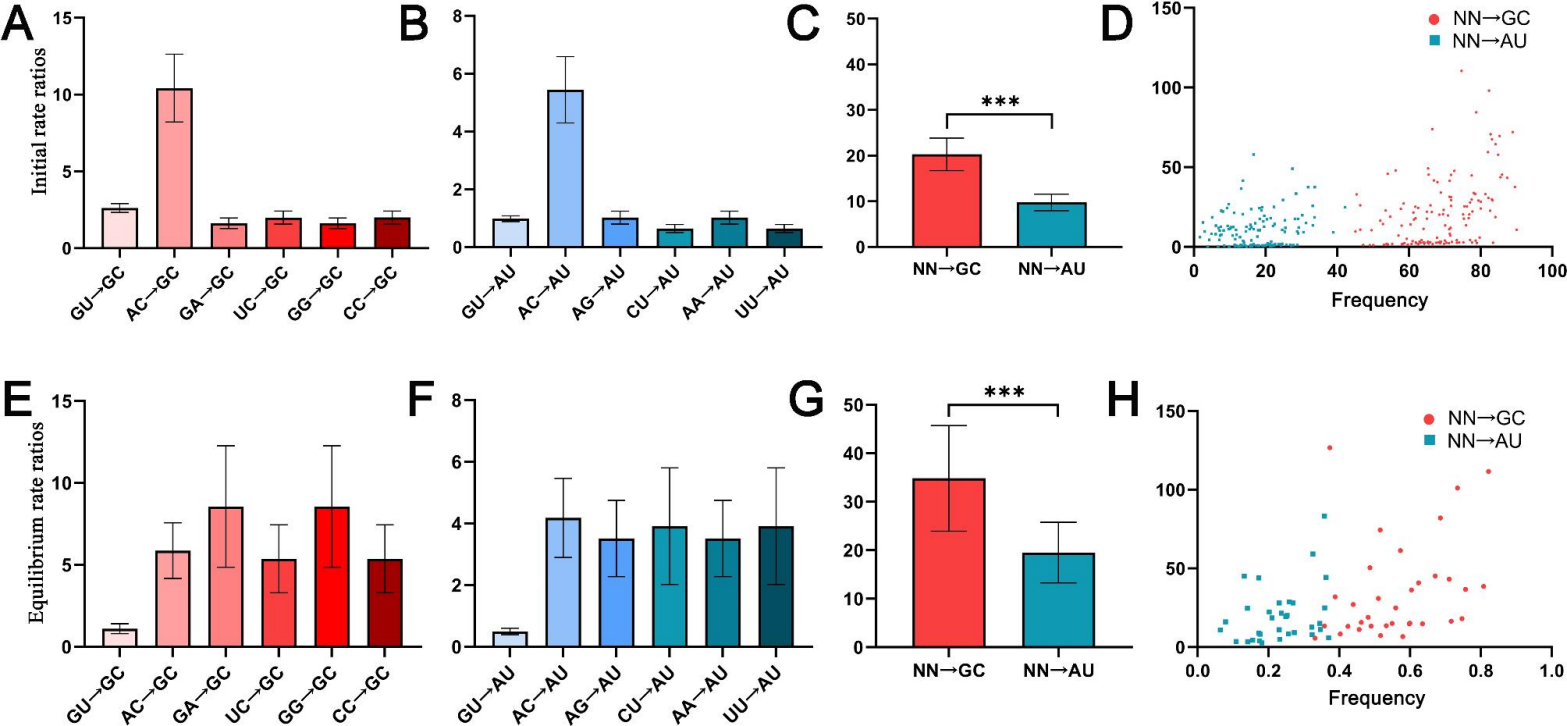
Comparison of base-pair transformations to GC and AU in ITS2 transition-rate matrices using the best-fit RNA substitution models. The transition rate for each matrix was normalized to an average substitution rate of 1.0. **A-D** Base-pair transformations derived from the initial states of 142 ITS2 transition-rate matrices, **A,B** The relative rates of the six possible transformations to GC and AU, respectively. **C** Comparison of the total formation rates for GC and AU base pairs. **D** Scatter plot showing the frequency-mutability relationship of formation rates for GC and AU base pairs, indicating an increased fixation of GC with GC enrichment. **E-H** base-pair transformations derived from the equilibrium states of 46 ITS2 transition-rate matrices. **E,F** The relative rates of the six possible transformations to GC and AU, respectively. **G** Comparison of the total formation rates for GC and AU base pairs. **H** Scatter plot illustrating the frequency-mutability relationship of formation rates for GC and AU base pairs, indicating an increased fixation of GC with GC enrichment

Using an evolutionary model, we can infer the substitution process, including base frequency and rate parameters, based on the basic assumption that the substitution process is constant within a given lineage. We found that the most common best-fit RNA substitution model was RNA16D (46.06%), followed by RNA16C (30.30%) and RNA7G (12.73%; Additional file 1: Table S1), none of which allows for simultaneous substitutions of both nucleotides in a base pair according to the model definition [33]. Alternatively, the base-pair substitution occurred mainly through double one-site substitutions by an intermediate state, i.e. AU-GU-GC. In total, there were six one-site substitutions from intermediates to GC and AU, respectively (Fig. 6). We compared the substitution rates among these six one-site substitutions in an initial tree (initial state) and found that these substitution rates were more or less equal, except for the extremely high rate of AC→GC (10.42), which accounted for 51% of all substitution rates for GC (Fig. 6A; Additional file 1: Table S2). Likewise, the rate of AC→AU (5.44) was the highest and accounted for 56% of all substitution rates to AU (Fig. 6B). Taken together, the GC generation rate was 208% higher than that of AU (Fig. 6C). Notably, we found that the GC generation rate was positively correlated with the GC frequency (*r* = 0.419, *P* < 10^− 6^). In other words, the fast mutation of GC base pairs lead to an increase in the probability of GC-allele fixation compared to that of the AT allele. When the substitution was expected at equilibrium, the rates of AC→GC and AC→AU were no longer significantly higher compared to other base-pair changes. Except for the GU→GC and GU→AU, the special change from relative stable to stable base pairs (Additional file 1: Table S3), the substitution rates from all unstable base pairs to stable base pairs (GC and AU) have increased in this state. On the whole, both the GC-generating rate and GC frequency were higher than those of AU. This elevated fixation of GC-enriching mutations is consistent with the above GC/K results, suggesting that gBGC might be a selective force driving GC content augmentation.

**Fig. 7:**
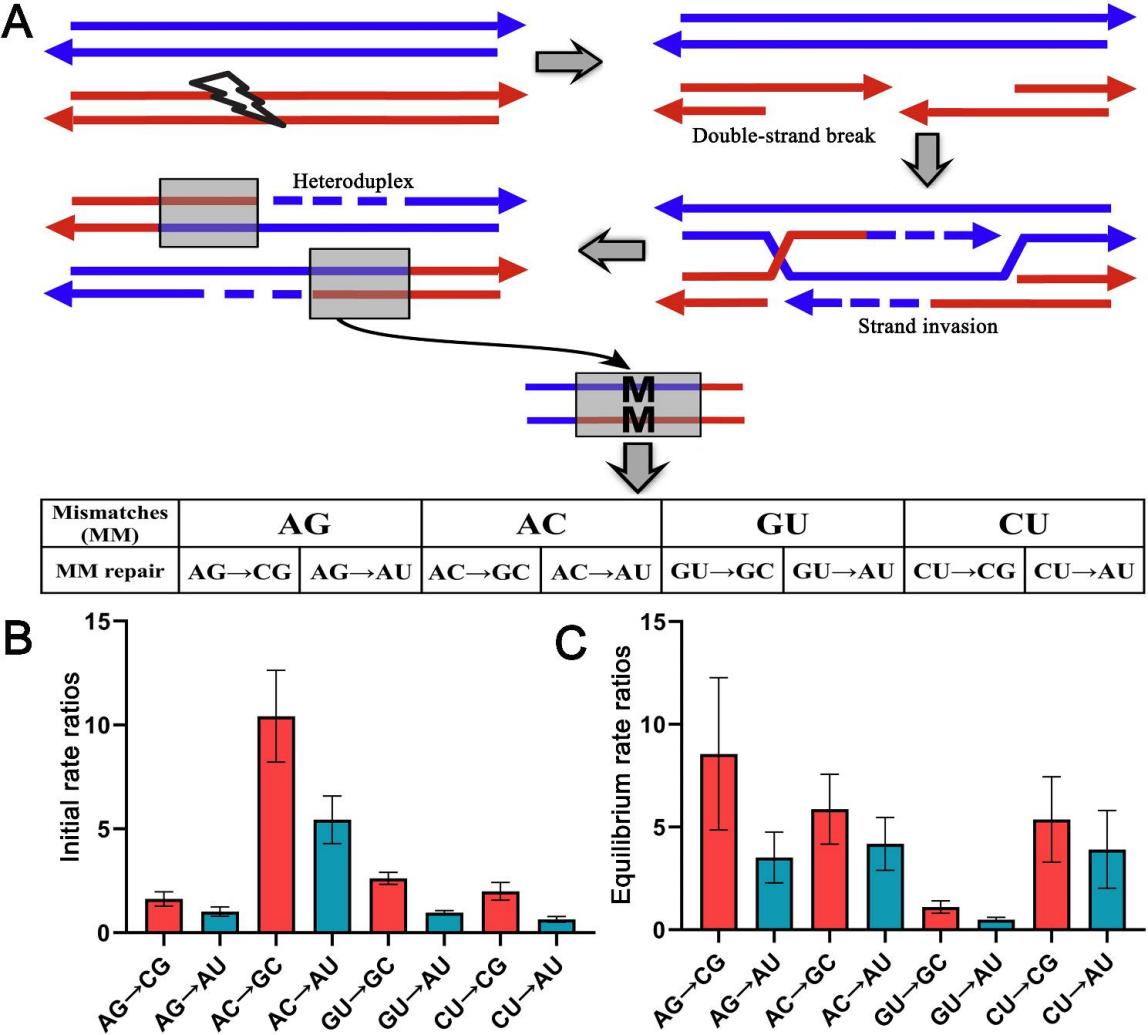
Mismatch base-pair transformation in meiotic recombination. **A** A schematic representation of gene conversion during meiotic recombination. The double-strand always break during meiosis, and a heteroduplex formed when the single-stranded DNA invades the homologous sequence, enabling the repair of up to four possible mismatches by altering one side of the nucleotides. **B,C** Comparison of mismatch base-pair transformations in both (B) initial and (C) equilibrium states reveals a GC-biased gene conversion

There were a total of four possible mismatched base pairs (MM: AG\AC\GU\CU) in the heterozygous sites after chromosome recombination, all of which can change into the stable AU or GC base pairs, e.g. AG→CG, AG→AU (Fig. 7A). Therefore, there are a total of eight (four pairs) base-pair changes in the heterozygous sites. We calculated the substitution rate among these four-pair substitutions in an initial state and found that different MMs had various substitution rates, among which the highest was AC→GC (42%) followed by the AC→AU (22%), which accounted for 64% of all eight possible base pair changes (Additional file 1: Table S4). Notably, substitution to GC was always higher than that to AU for each MM, averaging 206% that of AU (Fig. 7B). When the substitution was expected at equilibrium, the substitution rates of AC→GC and AC→AU were no longer higher due to the increase of other base-pair substitution rates, except for the relatively stable base-pair changes of GU→GC and GU→AU (Additional file 1: Table S5). However, the substitution to GC was still higher than that to AU for each MM, averaging 174% that of AU (Fig. 7C). Clearly, there was a MM conversion bias toward the GC base pair during the mismatch repair.

## Discussion

As one of the fundamental parameters of genome sequences, genomic GC content has been studied for more than a half-century, but its variability and underlying mechanisms remain unknown. Until recently, our knowledge of GC content and its evolutionary force was primarily derived from genome-wide or large-scale sequence analyses, such as the well-known feature of mammalian isochores shaped by the chromosome recombination and GC-biased gene conversion [4, 13, 21, 25]. Unlike animals, the plant genomes have always undergone polyploidization [34], making the relationship between recombination and GC-content more complex [22]. In the absence of the genomic sequence, the GC content of the third coding position (GC3) has traditionally utilized as a proxy for the GC composition of the isochore due to their synonymous nature and near neutrality or weak selection. Nonetheless, it is crucial to acknowledge that this association may not be valid in certain small-scale analyses [35, 36]. Herein, the GC-content study of rDNA ITS2 sequence-structure represents an alternative GC exploration at the local scale, which also sheds new light on the whole GC at the transcriptome level, since rRNA accounts for more than 60% of total cellular RNA. In addition, as ITS2 is a widely used marker in plant systematics, our findings on ITS2 GC content variation could greatly improve its evolutionary model and facilitate its phylogenetic use.

In contrast to the limited existing research on genomic-level variation mechanisms in GC, this study seeks to examine the patterns of ITS2 GC variation and the driving forces at the level of single genes. This investigation utilized the substantial availability of ITS2 sequences in the primary lineages of angiosperm phylogeny, comprising 8666 species and 63 families. We showed that the ITS2 GC content varies considerably within angiosperms, among which the grasses always have a higher ITS2 GC content. Furthermore, GC-rich ITS2 sequences always couple with GC heterogeneity. Both of these findings are consistent with the previous genome-wide GC investigations [10, 16]. Our additional correlation test indeed confirmed a positive association between the GC contents of ITS2 and the EST content of GC3 (Fig. 2B). This suggests that ITS2 can serve as a proxy for GC3 in inferring the genomic composition of the isochore in situations where a large-scale dataset of coding sequences is unavailable [37]. However, ITS2 GC content did not exhibit the same characteristic bimodal distribution as that of grass genomes [15, 16, 38]. Furthermore, our broad phylogenetic survey showed that ITS2 GC contents vary considerably within both the gymnosperm and the basal angiosperm (Fig. 1), contradicting the widely held belief that genome GC content evolves from the GC-poor and homogeneous ancestral genome to the GC-rich and highly heterogeneous grass genomes [16, 38]. Overall, ITS2 GC content varies similarly with the genome GC content among angiosperms but has its own unique evolutionary scenario.

The cleavage of ITS2 is a crucial step in the maturation of precursor ribosomal RNA (pre-rRNA) [39]. In our study, all the ITS2 sequences exhibited a highly conserved ‘four-fingered hand’ structure, which is a characteristic feature shared among angiosperms [26–28]. This indicates that there has been selective pressure on maintaining this functional secondary structure. A significant finding of our study is the uneven distribution of GC base pairs within the ITS2 secondary structure. Specifically the GC in ITS2 stems is significantly higher than that in loops (Fig. 3). This difference can be attributed to the fact that GC base pairs are more thermally stable due to their triple hydrogen bonding, as compared to AT base pairs. Our findings provide support for the hypothesis that functional rRNA sequences have been evolutionarily selected to enhance their structural and thermodynamic stability [40].

The thermal stability of GC-rich DNA leads to the assumption that the GC content of the genome is associated with environmental temperature [3, 4, 41]. However, our findings indicate that the thermal significance lies in the GC content of the double-stranded regions of structural RNAs rather than the entire sequence of transcriptome. Consistently, certain studies conducted on prokaryotic genomes have demonstrated an insignificance between the overall G + C content of the genome and the optimal temperature for bacterial growth, while a close relationship exists with the G + C content of structural RNAs [42, 43]. Taken together, our finding highlights that thermal adaptation potentially influences the composition of the double-stranded regions in structural RNAs rather than the overall nucleotide content of the genome.

The equilibrium GC content (GC*) represents the GC content at which a sequence evolves when substitution rates remain constant over time [11]. Therefore, GC* can provide insight into the latest trend in the evolution of GC content [21, 44]. Our observation indicates that in the GC-poor region (ITS2 unpaired region), GC* and GC are quite similar. Conversely in the GC-rich region (ITS2 paired region), both GC* and G-C base pair frequencies are significantly lower than the current GC content, implying that structure-related driving forces have maintained the current elevated GC content.

With the in-depth investigation of the unique isochore phenomenon, the gBGC mechanism has come to be regarded as the primary driving force for GC enrichment in mammalian genomes [21]. gBGC is regarded as a recombination-driven process that prefers GC over AT bases during the heteroduplex mismatch repair [20, 45]. Similarly, ITS2 alleles also undergo the recombination-associated concerted evolution, resulting in homologous sequences [29]. The shared recombination process between ITS2 alleles enable us to test whether the increase in ITS2 GC coincides with a decrease in ITS2 polymorphism. The average number of nucleotide differences (K) in ITS2 decreased with GC enrichment (Fig. 5A-C), providing support for gBGC’s role in ITS2. However, it is not the case for the ITS2 unpaired region, where the GC enrichment is uncorrelated with ITS2 homogenization. This suggests that gBGC alone cannot fully explain the observed overall ITS2 GC enrichment in this study. In addition, we found that molecular substitutions in paired regions differed strikingly from those in unpaired regions. In paired regions, the best-fit RNA substitution model of the ITS2 transition-rate matrix showed a higher rate of base-pair transformations to GC than transformations to AU. Notably, these increased GC transformation rates have promoted GC fixations, similar to positively selected mutation. An extensive study of base-pair mismatch transformations showed that for all eight possible mismatch repair pathways, mismatch transformations to GC/CG were significantly higher than transformations to AU/UA. These results provide compelling evidence to support the gBGC hypothesis that transformation favors GC over AT during heteroduplex repair in recombination [20, 45].

## Conclusions

Traditional studies of GC content always focus on the genomic scale, leaving an open question of how GC varies at single gene scale. This study promotes the understanding of GC variation at the local genome region by using the ITS2 regions, a most widely used marker in plant systematics. The availability of volume sequences allows us to demonstrate that GC-biased gene conversion (gBGC) occurs in conjunction with concerted evolution in ITS2, resulting in the enrichment of GC in certain homogeneous sequences. Furthermore, the RNA substitution models utilized in this study support the presence of gBGC, as the transformation probabilities of GC base pairs were significantly higher compared to AU base pairs. The secondary structure of ITS2 is recognized as a “four-fingered hand” and our findings reveal that the stem GC content is notably higher than that of the loops. This observation aligns with the hypothesis of thermodynamic stability. Taken together, we hypothesize that gBGC contributes to an increase in GC content, while the ITS2 secondary structure enhances GC selection. To test this assumption, it would be necessary to investigate the equilibrium GC content in the absence of current secondary structure constraints. As anticipated, the GC content in unpaired regions remains relatively constant, whereas the GC content in paired regions exhibits a significant decrease. This finding underscores the importance of secondary structure in maintaining the current enrichment of GC. In conclusion, this study sheds light on the variability of ITS2 GC content within sequences and lineages and emphasizes the role of secondary structure when examining GC content at the local RNA scale.

## Methods

### Taxon sampling and ITS2 sequence acquisition

We sampled plant lineages from the NCBI database (accessed on May 2022), for which ribosomal ITS/ITS2 sequences are available from closely related species. We focused on lineages that presented DNA barcodes, as they generally have adequate inter- and intraspecific sampling for effective species identification. The validity and coverage of species within a given lineage were investigated using the Plant List online service (http://www.theplantlist.org). Lineage representativeness among the major angiosperms was also assessed based on the Angiosperm Phylogeny Group IV system (APG IV, 2016) [46]. All sequences of these lineages with the annotation “internal transcribed spacer” or “internal transcribed spacer 2” were selected. Then, ITS2 boundaries were determined by using GenBank annotations or the hidden Markov models implemented in the ITS2 database (http://its2.bioapps.biozentrum.uniwuerzburg.de/) [47]. All sequences of each genus were aligned and edited by BioEdit (with default parameters) [48], where incomplete ITS2 sequences were excluded. A total of 8666 species representing 165 genera, 63 families, and 30 orders were finally selected for analyses. We also retrieved some EST unigene datasets from Serres-Giardi’s previous study [15] and tested the correlation of GC contents between the EST and ITS2 among the shared genera.

### ITS2 secondary-structure prediction and partition

The secondary structure of individual ITS2 (Vienna format) was predicted using homology prediction with the most similar sequence to a model structure in the database [49]. Next, the sequence structures of the whole genus were aligned synchronously using 4SALE 1.7 (with default parameters) [50] to obtain a consensus secondary structure. Using this consensus secondary structure, the primary sequence of ITS2 was partitioned into paired and unpaired regions. The sequence logo, which is generated on the LogoJS website (https://logojs.wenglab.org/app/), graphically represents the relative frequency of bases at each position in the consensus secondary structure. The GC content of the whole ITS2 and its partitioned regions were calculated using MEGA 7 [51].

### Inferring substitution parameters of ITS2 sequence structure

Given that the paired and unpaired regions may have different evolutionary patterns, a RNA-specific Perl script (model_selection.pl) from PHASE package 3.0 [52] were used to infer ITS2 substitution. This Perl script includes two DNA models (HKY85 and REV) for unpaired regions, seven RNA 7-state models, and nine RNA 16-state models for paired regions. Allen and Whelan’s likelihood-correction method was used to account for the different numbers of parameters between the four-, seven-, and 16-state models and thus facilitate comparison between the 4-, 7-, and 16-state models [52]. The best-fit mixed models were selected according to the corrected version of Akaike’s information criterion (AICc). Based on the best-fit mixed model, the phylogenetic analyses were performed using the mcmcPHASE program from the PHASE package. The MCMC analysis was run for 20 million generations, with four separate chains, starting from a ML tree topology, and retaining one out of every 100 generations. The first 3000 trees were burned-in, and the remaining trees were used to calculate the majority-rule-consensus topology and posterior probabilities by the mcmcsummarize program in the PHASE package. We found that matrixes with fewer species or few site variations hardly reached convergence; a total of 53 data sets were verified at convergence by using Tracer 1.6. For each data set, substitution-rate parameter values and GC base-pair frequencies at equilibrium were generated using the mcmcsummarize program from the PHASE package.

### ITS2 sequence diversity and GC content

Both the ITS2 concerted evolution and the gBGC are driven by recombination. We thus hypothesize that ITS2 homogenization couple with GC proliferation. To test this idea, the average number of ITS2 nucleotide differences (K) of each genus was calculated using DnaSP 6 [53]. Then the GC content of all paired and unpaired regions were calculated separately using MEGA.

### Phylogenetic annotation of GC content

To explore how GC variation occurs among angiosperms, we plot the GC content of each genus in a phylogenetic context. As ITS2 was too short to resolve most of the generic relationships, we alternatively constructed a phylogenetic tree of all our genera by using the online service of the NCBI taxonomy common tree (https://www.ncbi.nlm.nih.gov/Taxonomy/CommonTree/wwwcmt.cgi). This tree is consistent with the APG IV [46] and provided well-supported relationships among the plant orders, despite the fact that some relationships at low taxonomic levels were still unresolved. This phylogenetic tree in phylip format was imported into the online tool Interactive Tree Of Life (ITOL: https://itol.embl.de/), wherein the tree was edited and annotated with GC content.

### Data analysis

We utilized SPSS 22.0 and GraphPad Prism 8 software for statistical analyses in order to summarize the results. To assess the correlation between EST-GC3 and ITS2 GC, we conducted Spearman’s correlation tests. Spearman’s correlation test was chosen due to the deviation from normal distribution in the EST-GC3 dataset. Pearson’s correlation test was selected for all other analyses, as it is more appropriate for larger sample sizes and normally distributed data in this particular study. This choice ensures the prevention of potential information loss, which can occur with the application of Spearman’s correlation test. Independent-Samples t-tests were employed to evaluate the differences in data.

### Electronic supplementary material

Below is the link to the electronic supplementary material.

Supplementary Material 1

## Data Availability

The ITS2 sequence data and the sequence-structure alignment of each sample are posted at: https://figshare.com/articles/dataset/GC_heterogeneity_reveals_sequence-structures_evolution_of_angiosperm_ITS2/22178237.
