# Supplementary material for: GC heterogeneity reveals sequence-structures evolution of angiosperm ITS2
Source: BMC Plant Biol. 2023 Dec 1;23:608. doi: 10.1186/s12870-023-04634-9 (PMC10691020; doi:10.1186/s12870-023-04634-9)
Supplement: Supplementary file 1 — Supplementary Material 1 [file 12870_2023_4634_MOESM1_ESM.docx]

Table S1. Taxonomy, number of species, ITS2 sequence-structure evolution and their GC content

| Order | Family | Genus | # of species | K value | | | GC content (%)^a^ | | | Best-fit models | Equilibrium GC content (GC*; %) | | | |
| --- | --- | --- | --- | --- | --- | --- | --- | --- | --- | --- | --- | --- | --- | --- |
|  |  |  |  | Total K | Paired K | Unpaired K | Mean | GC_p_ | GC_up_ |  | Mean | GC^*^_p_ | GC^*^_up_ | G-C frequency |
| Alismatales | Alismataceae | *Sagittaria* | 17 | 22.382 | 12.926 | 9.456 | 55.44 | 66.38 | 38.37 | HKY85+G_RNA16D | 51.75 | 60.88 | 37.50 | 49.64 |
| Apiales | Araliaceae | *Eleutherococcus* | 28 | 6.356 | 3.289 | 3.067 | 62.59 | 70.65 | 49.78 | HKY85+G_RNA16C | 61.45 | 69.55 | 48.57 | 59.93 |
| Apiales | Araliaceae | *Hedera* | 42 | 6.364 | 4.545 | 1.819 | 61.43 | 69.35 | 49.67 | REV+G_RNA16C | 56.86 | 63.07 | 47.63 | 55.59 |
| Apiales | Araliaceae | *Schefflera* | 64 | 18.019 | 7.507 | 10.512 | 62.12 | 73.12 | 46.75 | HKY85+G_RNA16C | 58.51 | 67.30 | 46.24 | 55.54 |
| Asparagales | Amaryllidaceae | *Allium* | 27 | 23.732 | 12.439 | 11.293 | 47.45 | 51.75 | 39.54 | HKY85+G_RNA16D | 44.10 | 47.39 | 38.04 | 33.22 |
| Asparagales | Iridaceae | *Crocus* | 11 | 12.745 | 2.000 | 10.745 | 65.72 | 74.55 | 55.27 | HKY85+G_RNA16D | 62.33 | 68.48 | 55.06 | 67.14 |
| Asterales | Asteraceae | *Carthamus* | 31 | 6.942 | 2.830 | 4.112 | 56.26 | 65.05 | 41.23 | HKY85+G_RNA16D | 52.11 | 57.89 | 42.22 | 43.96 |
| Asterales | Asteraceae | *Gaillardia* | 40 | 13.221 | 8.265 | 4.956 | 50.65 | 60.19 | 34.14 | REV+G_RNA16C | 49.60 | 56.41 | 37.81 | 39.68 |
| Asterales | Asteraceae | *Helianthus* | 50 | 2.656 | 1.817 | 0.838 | 54.31 | 64.58 | 31.39 | REV+G_RNA16D | 46.90 | 51.01 | 37.73 | 48.20 |
| Asterales | Asteraceae | *Lactuca* | 29 | 22.140 | 9.921 | 12.219 | 53.79 | 64.40 | 39.18 | HKY85+G_RNA16D | 41.72 | 48.33 | 32.62 | 35.88 |
| Asterales | Asteraceae | *Lasthenia* | 54 | 23.928 | 12.758 | 11.170 | 47.90 | 60.11 | 25.26 | REV+G_RNA16C | 45.06 | 56.79 | 23.32 | 43.32 |
| Asterales | Asteraceae | *Sinosenecio* | 64 | 19.868 | 10.316 | 9.552 | 52.97 | 64.92 | 34.61 | REV+G_RNA16C | 49.09 | 60.28 | 31.88 | 46.94 |
| Brassicales | Brassicaceae | *Arabidopsis* | 14 | 11.484 | 4.802 | 6.681 | 55.84 | 65.13 | 44.05 | REV+G_RNA16D | 52.75 | 60.59 | 42.79 | 49.00 |
| Brassicales | Brassicaceae | *Noccaea* | 49 | 5.007 | 1.568 | 3.439 | 52.58 | 61.49 | 39.74 | HKY85+G_RNA16D | 51.78 | 61.50 | 37.80 | 37.34 |
| Caryophyllales | Amaranthaceae | *Beta* | 7 | 6.381 | 1.048 | 5.333 | 62.64 | 69.25 | 53.69 | HKY85+G_RNA7G | 59.69 | 69.03 | 47.01 | 61.96 |
| Cucurbitales | Cucurbitaceae | *Citrullus* | 8 | 8.500 | 3.500 | 5.000 | 65.70 | 74.94 | 52.73 | HKY85+G_RNA7G | 69.62 | 76.21 | 60.36 | 71.37 |
| Cucurbitales | Cucurbitaceae | *Cucumis* | 36 | 10.860 | 3.316 | 7.544 | 64.63 | 73.29 | 53.48 | REV+G_RNA16K | 56.57 | 57.39 | 55.52 | 62.44 |
| Ericales | Ericaceae | *Rhododendron* | 126 | 9.270 | 2.191 | 7.079 | 57.48 | 65.67 | 45.44 | HKY85+G_RNA16D | 52.16 | 56.40 | 45.93 | 55.22 |
| Ericales | Primulaceae | *Cyclamen* | 19 | 21.216 | 4.836 | 16.380 | 67.37 | 76.08 | 60.78 | REV+G_RNA16C | 67.43 | 79.57 | 58.24 | 74.72 |
| Fabales | Fabaceae | *Arachis* | 48 | 8.171 | 2.949 | 5.222 | 72.21 | 78.24 | 61.33 | REV+G_RNA16D | 64.71 | 64.34 | 65.38 | 59.96 |
| Fabales | Fabaceae | *Cicer* | 28 | 3.344 | 1.788 | 1.556 | 48.86 | 56.70 | 32.94 | HKY85+G_RNA16D | 43.62 | 46.62 | 37.55 | 38.87 |
| Fabales | Fabaceae | *Cyamopsis* | 4 | 17.000 | 8.667 | 8.333 | 49.88 | 55.30 | 41.36 | HKY85+G_RNA16D | 49.08 | 52.89 | 43.08 | 40.30 |
| Fabales | Fabaceae | *Glycine* | 20 | 11.179 | 4.442 | 6.737 | 59.22 | 63.05 | 53.40 | REV+G_RNA16D | 58.95 | 58.39 | 59.80 | 53.32 |
| Fagales | Fagaceae | *Castanea* | 6 | 3.867 | 0.667 | 3.200 | 72.87 | 73.92 | 71.00 | HKY85+G_RNA7G | 73.68 | 72.39 | 75.98 | 66.03 |
| Fagales | Fagaceae | *Fagus* | 10 | 6.178 | 1.756 | 4.422 | 65.52 | 71.99 | 54.64 | REV+G_RNA7G | 64.79 | 71.15 | 54.10 | 61.46 |
| Lamiales | Lamiaceae | *Ocimum* | 4 | 21.000 | 6.500 | 14.500 | 71.56 | 78.44 | 63.85 | HKY85+G_RNA7G | 72.92 | 80.99 | 63.90 | 73.95 |
| Lamiales | Lamiaceae | *Salvia* | 60 | 18.907 | 11.544 | 7.363 | 66.57 | 79.72 | 46.56 | HKY85+G_RNA16C | 63.24 | 72.77 | 48.74 | 62.36 |
| Lamiales | Plantaginaceae | *Antirrhinum* | 38 | 6.212 | 2.690 | 3.522 | 63.09 | 69.92 | 50.57 | HKY85+G_RNA16D | 62.37 | 70.81 | 46.89 | 60.40 |
| Laurales | Lauraceae | *Persea* | 33 | 7.174 | 3.542 | 3.632 | 77.11 | 84.85 | 63.19 | REV+G_RNA16D | 73.68 | 76.91 | 67.88 | 80.82 |
| Malpighiales | Euphorbiaceae | *Euphorbia* | 9 | 27.528 | 6.667 | 20.861 | 61.51 | 78.25 | 46.95 | HKY85+G_RNA16C | 61.29 | 80.36 | 44.70 | 71.65 |
| Malpighiales | Rhizophoraceae | *Bruguiera* | 5 | 2.800 | 1.800 | 1.000 | 72.40 | 81.25 | 59.63 | HKY85+G_RNA7G | 73.67 | 82.12 | 61.47 | 74.08 |
| Piperales | Aristolochiaceae | *Asarum* | 38 | 9.218 | 3.821 | 5.397 | 52.76 | 60.57 | 38.61 | REV+G_RNA16D | 58.35 | 69.10 | 38.89 | 50.56 |
| Poales | Poaceae | *Aegilops* | 22 | 3.814 | 1.662 | 2.152 | 63.67 | 79.05 | 42.28 | REV+G_RNA16D | 57.33 | 69.41 | 40.53 | 73.44 |
| Poales | Poaceae | *Agrostis* | 48 | 8.160 | 3.269 | 4.892 | 65.96 | 79.57 | 54.21 | REV+G_RNA16C | 60.26 | 71.14 | 50.86 | 59.80 |
| Poales | Poaceae | *Eleusine* | 9 | 11.694 | 2.889 | 8.806 | 52.04 | 58.00 | 44.36 | HKY85+G_RNA16D | 48.36 | 54.88 | 39.96 | 51.06 |
| Poales | Poaceae | *Hordeum* | 29 | 15.485 | 7.562 | 7.924 | 60.70 | 73.09 | 41.32 | REV+G_RNA16D | 53.80 | 62.07 | 40.86 | 51.72 |
| Poales | Poaceae | *Oryza* | 17 | 9.978 | 3.074 | 6.904 | 76.98 | 87.92 | 59.29 | REV+G_RNA16C | 78.16 | 87.15 | 63.63 | 82.22 |
| Poales | Poaceae | *Pennisetum* | 13 | 18.526 | 5.936 | 12.590 | 60.40 | 66.93 | 52.36 | HKY85+G_RNA16D | 57.11 | 61.43 | 51.78 | 54.96 |
| Poales | Poaceae | *Setaria* | 16 | 22.533 | 8.542 | 13.991 | 59.87 | 71.54 | 38.78 | HKY85+G_RNA16C | 60.13 | 73.11 | 36.68 | 63.53 |
| Poales | Poaceae | *Sorghum* | 22 | 10.918 | 3.026 | 7.892 | 68.80 | 75.24 | 57.81 | REV+G_RNA16D | 62.19 | 63.25 | 60.39 | 68.58 |
| Ranunculales | Papaveraceae | *Eschscholzia* | 12 | 15.530 | 3.742 | 11.788 | 71.69 | 83.97 | 54.97 | REV+G_RNA16C | 71.59 | 82.41 | 56.86 | 75.70 |
| Ranunculales | Ranunculaceae | *Adonis* | 12 | 8.136 | 3.439 | 5.697 | 52.99 | 60.01 | 39.98 | HKY85+G_RNA16D | 51.26 | 56.25 | 42.03 | 51.58 |
| Rosales | Rosaceae | *Fragaria* | 17 | 4.478 | 1.059 | 3.419 | 65.81 | 73.58 | 53.27 | REV+G_RNA16D | 62.12 | 65.45 | 56.73 | 71.06 |
| Rosales | Rosaceae | *Malus* | 32 | 13.006 | 6.524 | 6.482 | 70.75 | 75.34 | 61.47 | REV+G_RNA16D | 67.44 | 67.10 | 68.14 | 57.96 |
| Rosales | Rosaceae | *Rosa* | 68 | 1.636 | 0.411 | 1.225 | 57.75 | 67.35 | 41.81 | HKY85+G_RNA16D | 64.16 | 77.30 | 42.34 | 48.70 |
| Sapindales | Meliaceae | *Cedrela* | 25 | 8.677 | 3.630 | 5.047 | 74.23 | 82.96 | 60.23 | REV+G_RNA16C | 69.74 | 76.95 | 58.18 | 71.36 |
| Saxifragales | Crassulaceae | *Sedum* | 100 | 36.089 | 20.194 | 15.895 | 54.82 | 65.66 | 42.82 | HKY85+G_RNA16C | 50.88 | 58.90 | 42.01 | 43.58 |
| Saxifragales | Grossulariaceae | *Ribes* | 80 | 4.281 | 1.901 | 2.380 | 58.19 | 65.50 | 48.45 | REV+G_RNA16D | 49.99 | 52.91 | 46.09 | 42.46 |
| Saxifragales | Paeoniaceae | *Paeonia* | 38 | 2.559 | 1.506 | 1.053 | 58.19 | 66.66 | 44.78 | REV+G_RNA16C | 55.10 | 64.50 | 40.20 | 55.98 |
| Solanales | Convolvulaceae | *Calystegia* | 37 | 2.636 | 0.182 | 2.455 | 66.47 | 77.41 | 51.15 | HKY85+G_RNA7G | 64.47 | 73.12 | 52.37 | 62.84 |
| Solanales | Solanaceae | *Petunia* | 15 | 1.943 | 1.010 | 0.933 | 60.22 | 68.88 | 46.62 | REV+G_RNA16C | 62.02 | 68.54 | 51.79 | 57.33 |
| Zingiberales | Musaceae | *Musa* | 56 | 8.084 | 4.384 | 3.700 | 69.98 | 71.88 | 66.16 | REV+G_RNA16C | 63.79 | 63.74 | 63.88 | 45.64 |
| Zingiberales | Zingiberaceae | *Curcuma* | 52 | 8.833 | 4.396 | 4.437 | 57.05 | 61.57 | 50.84 | HKY85+G_RNA16D | 54.06 | 57.18 | 49.78 | 46.24 |
| Acorales | Acoraceae | *Acorus* | 7 | 13.286 | 3.905 | 9.381 | 72.10 | 83.01 | 60.39 | HKY85+G_RNA16D | Premature Convergence | | | |
| Apiales | Apiaceae | *Angelica* | 65 | 13.870 | 5.778 | 8.092 | 56.41 | 69.42 | 37.35 | HKY85+G_RNA16D |  |  |  |  |
| Apiales | Apiaceae | *Apium* | 10 | 4.400 | 2.756 | 1.644 | 52.82 | 64.42 | 33.80 | HKY85+G_RNA16C |  |  |  |  |
| Apiales | Apiaceae | *Bupleurum* | 63 | 14.749 | 6.936 | 7.813 | 58.88 | 70.61 | 43.24 | HKY85+G_RNA16C | Premature Convergence | | | |
| Apiales | Araliaceae | *Aralia* | 60 | 7.802 | 4.818 | 2.984 | 63.82 | 73.58 | 47.28 | REV+G_RNA16D |  |  |  |  |
| Apiales | Araliaceae | *Brassaiopsis* | 26 | 7.023 | 3.485 | 3.538 | 62.01 | 73.53 | 44.82 | HKY85+G_RNA7G |  |  |  |  |
| Apiales | Araliaceae | *Dendropanax* | 52 | 12.724 | 6.490 | 6.234 | 63.38 | 72.51 | 48.63 | REV+G_RNA16D |  |  |  |  |
| Apiales | Araliaceae | *Macropanax* | 13 | 9.500 | 5.000 | 4.500 | 63.65 | 73.24 | 48.02 | REV+G_RNA16C |  |  |  |  |
| Apiales | Araliaceae | *Oplopanax* | 6 | 5.333 | 4.000 | 1.333 | 63.75 | 72.45 | 48.65 | HKY85+G_RNA16C |  |  |  |  |
| Apiales | Araliaceae | *Panax* | 17 | 8.699 | 4.471 | 4.228 | 64.29 | 72.33 | 50.14 | HKY85+G_RNA16C |  |  |  |  |
| Asparagales | Asparagaceae | *Asparagus* | 23 | 12.628 | 5.964 | 6.494 | 64.90 | 76.71 | 43.41 | REV+G_RNA16A |  |  |  |  |
| Asparagales | Iridaceae | *Iris* | 17 | 25.551 | 10.934 | 14.617 | 69.17 | 77.08 | 56.13 | HKY85+G_RNA16J |  |  |  |  |
| Asparagales | Orchidaceae | *Habenaria* | 120 | 6.141 | 3.818 | 2.323 | 46.25 | 51.18 | 36.01 | REV+G_RNA16D |  |  |  |  |
| Asparagales | Orchidaceae | *Holcoglossum* | 55 | 5.926 | 2.581 | 3.345 | 69.13 | 76.52 | 54.69 | HKY85+G_RNA16D |  |  |  |  |
| Asparagales | Orchidaceae | *Phalaenopsis* | 42 | 17.934 | 9.800 | 8.134 | 72.48 | 77.21 | 63.12 | HKY85+G_RNA16D |  |  |  |  |
| Asterales | Asteraceae | *Barnadesia* | 9 | 14.444 | 6.528 | 7.917 | 64.61 | 64.90 | 64.14 | HKY85+G_RNA16D |  |  |  |  |
| Asterales | Asteraceae | *Chaetanthera* | 82 | 27.376 | 13.693 | 13.683 | 59.15 | 74.64 | 39.66 | REV+G_RNA16D |  |  |  |  |
| Asterales | Asteraceae | *Chrysanthemum* | 34 | 3.010 | 1.648 | 1.362 | 52.99 | 64.80 | 33.74 | HKY85+G_RNA16D |  |  |  |  |
| Asterales | Asteraceae | *Cichorium* | 7 | 0.857 | 0.857 | 0.000 | 58.34 | 72.23 | 36.48 | HKY85+G_RNA7G |  |  |  |  |
| Asterales | Asteraceae | *Cynara* | 11 | 3.491 | 0.982 | 2.509 | 63.64 | 69.13 | 55.24 | HKY85+G_RNA16C |  |  |  |  |
| Asterales | Asteraceae | *Dahlia* | 47 | 9.571 | 5.433 | 4.138 | 56.77 | 66.11 | 35.94 | HKY85+G_RNA16D |  |  |  |  |
| Asterales | Asteraceae | *Flaveria* | 67 | 18.237 | 10.674 | 7.563 | 46.24 | 56.07 | 23.98 | HKY85+G_RNA16D |  |  |  |  |
| Asterales | Asteraceae | *Lessingia* | 38 | 3.264 | 1.813 | 1.451 | 50.00 | 55.67 | 38.47 | HKY85+G_RNA16D |  |  |  |  |
| Asterales | Asteraceae | *Melampodium* | 83 | 26.810 | 13.935 | 12.875 | 53.13 | 64.71 | 34.23 | REV+G_RNA16D |  |  |  |  |
| Asterales | Asteraceae | *Senecio* | 23 | 7.534 | 3.067 | 4.467 | 50.91 | 61.49 | 35.14 | HKY85+G_RNA16C |  |  |  |  |
| Asterales | Asteraceae | *Soroseris* | 19 | 1.848 | 1.532 | 0.316 | 54.89 | 67.90 | 32.70 | HKY85+G_RNA7G |  |  |  |  |
| Asterales | Asteraceae | *Stevia* | 78 | 12.084 | 6.574 | 5.510 | 50.64 | 58.64 | 36.54 | HKY85+G_RNA16D |  |  |  |  |
| Asterales | Asteraceae | *Taraxacum* | 84 | 6.616 | 3.878 | 2.738 | 52.58 | 64.43 | 31.66 | HKY85+G_RNA16C |  |  |  |  |
| Asterales | Asteraceae | *Zinnia* | 14 | 20.231 | 8.637 | 11.594 | 52.15 | 60.58 | 38.25 | HKY85+G_RNA16C |  |  |  |  |
| Brassicales | Brassicaceae | *Brassica* | 23 | 11.901 | 5.498 | 6.403 | 54.60 | 67.57 | 36.54 | HKY85+G_RNA16C |  |  |  |  |
| Brassicales | Brassicaceae | *Draba* | 122 | 2.600 | 1.269 | 1.331 | 53.48 | 67.64 | 25.75 | HKY85+G_RNA16D |  |  |  |  |
| Brassicales | Brassicaceae | *Thellungiella* | 3 | 11.333 | 2.000 | 9.333 | 56.08 | 69.81 | 39.15 | REV+G_RNA7G |  |  |  |  |
| Caryophyllales | Aizoaceae | *Mesembryanthemum* | 7 | 10.429 | 2.857 | 7.572 | 71.73 | 85.07 | 59.54 | HKY85+G_RNA16C |  |  |  |  |
| Caryophyllales | Caryophyllaceae | *Silene* | 454 | 1.558 | 1.404 | 0.154 | 62.56 | 72.31 | 49.65 | REV+G_RNA16A |  |  |  |  |
| Caryophyllales | Plumbaginaceae | *Limonium* | 142 | 16.210 | 3.925 | 12.285 | 50.11 | 54.70 | 45.16 | REV+G_RNA16D |  |  |  |  |
| Caryophyllales | Polygonaceae | *Persicaria* | 63 | 17.923 | 10.062 | 7.861 | 67.63 | 76.89 | 50.48 | HKY85+G_RNA16D | Premature Convergence | | | |
| Caryophyllales | Tamaricaceae | *Tamarix* | 39 | 5.538 | 2.130 | 3.408 | 65.38 | 76.60 | 42.90 | REV+G_RNA16C |  |  |  |  |
| Celastrales | Celastraceae | *Euonymus* | 56 | 10.844 | 2.579 | 8.266 | 68.33 | 81.24 | 54.67 | REV+G_RNA16A |  |  |  |  |
| Celastrales | Parnassiaceae | *Parnassia* | 91 | 21.082 | 11.123 | 9.959 | 49.28 | 58.41 | 38.22 | REV+G_RNA16D |  |  |  |  |
| Cucurbitales | Cucurbitaceae | *Hemsleya* | 69 | 2.509 | 1.006 | 1.503 | 69.02 | 74.83 | 60.50 | REV+G_RNA16C |  |  |  |  |
| Dipsacales | Adoxaceae | *Viburnum* | 114 | 9.241 | 5.042 | 4.199 | 64.72 | 74.22 | 42.10 | REV+G_RNA16A |  |  |  |  |
| Ericales | Actinidiaceae | *Actinidia* | 38 | 15.309 | 6.862 | 8.447 | 56.16 | 59.50 | 51.69 | REV+G_RNA16D |  |  |  |  |
| Ericales | Ebenaceae | *Diospyros* | 36 | 16.651 | 5.313 | 11.338 | 77.35 | 87.19 | 66.90 | REV+G_RNA16D |  |  |  |  |
| Ericales | Ericaceae | *Enkianthus* | 16 | 13.000 | 6.167 | 6.833 | 66.68 | 73.12 | 53.38 | HKY85+G_RNA16C |  |  |  |  |
| Ericales | Ericaceae | *Vaccinium* | 108 | 7.450 | 3.621 | 3.829 | 63.74 | 73.61 | 46.47 | REV+G_RNA16D |  |  |  |  |
| Ericales | Primulaceae | *Lysimachia* | 87 | 27.731 | 11.040 | 16.691 | 59.87 | 74.81 | 39.47 | REV+G_RNA16C |  |  |  |  |
| Fabales | Fabaceae | *Astragalus* | 93 | 11.014 | 8.121 | 2.893 | 50.91 | 60.62 | 29.78 | HKY85+G_RNA16D |  |  |  |  |
| Fabales | Fabaceae | *Glycyrrhiza* | 6 | 5.800 | 2.533 | 3.267 | 53.72 | 57.02 | 47.85 | HKY85+G_RNA7G |  |  |  |  |
| Fabales | Fabaceae | *Lathyrus* | 91 | 3.901 | 2.435 | 1.466 | 47.36 | 64.40 | 39.18 | REV+G_RNA16D |  |  |  |  |
| Fabales | Fabaceae | *Lotus* | 142 | 12.560 | 4.635 | 7.925 | 52.23 | 60.33 | 43.61 | REV+G_RNA16D |  |  |  |  |
| Fabales | Fabaceae | *Lupinus* | 130 | 4.317 | 2.680 | 1.637 | 60.32 | 69.15 | 35.60 | HKY85+G_RNA16C |  |  |  |  |
| Fabales | Fabaceae | *Medicago* | 82 | 9.098 | 3.456 | 5.642 | 47.18 | 56.66 | 31.39 | REV+G_RNA16D |  |  |  |  |
| Fabales | Fabaceae | *Phaseolus* | 43 | 17.534 | 5.590 | 11.944 | 57.30 | 64.35 | 48.14 | HKY85+G_RNA16D |  |  |  |  |
| Fabales | Fabaceae | *Pisum* | 3 | 4.000 | 0.667 | 3.333 | 46.48 | 52.48 | 32.82 | HKY85+G_RNA7G |  |  |  |  |
| Fabales | Fabaceae | *Robinia* | 4 | 2.500 | 0.500 | 2.000 | 53.01 | 57.60 | 44.12 | HKY85+G_RNA7G |  |  |  |  |
| Fabales | Fabaceae | *Trifolium* | 220 | 9.823 | 5.588 | 4.235 | 47.06 | 56.52 | 32.42 | HKY85+G_RNA16D |  |  |  |  |
| Fabales | Fabaceae | *Vicia* | 142 | 6.085 | 2.918 | 3.167 | 47.56 | 57.19 | 32.53 | HKY85+G_RNA16D |  |  |  |  |
| Fabales | Fabaceae | *Vigna* | 12 | 41.167 | 8.227 | 32.940 | 50.41 | 56.58 | 47.43 | REV+G_RNA16D |  |  |  |  |
| Fagales | Betulaceae | *Betula* | 60 | 3.407 | 1.496 | 1.911 | 63.19 | 75.13 | 45.03 | HKY85+G_RNA16C |  |  |  |  |
| Fagales | Fagaceae | *Quercus* | 151 | 5.962 | 2.434 | 3.528 | 68.91 | 72.14 | 64.19 | REV+G_RNA16D |  |  |  |  |
| Fagales | Juglandaceae | *Juglans* | 23 | 7.858 | 2.087 | 5.771 | 58.26 | 72.91 | 37.52 | HKY85+G_RNA16C |  |  |  |  |
| Gentianales | Gentianaceae | *Swertia* | 73 | 16.038 | 9.666 | 6.372 | 61.50 | 71.06 | 47.42 | REV+G_RNA16D |  |  |  |  |
| Gentianales | Rubiaceae | *Coffea* | 41 | 5.804 | 1.435 | 4.368 | 69.51 | 76.41 | 58.35 | HKY85+G_RNA16C |  |  |  |  |
| Gentianales | Rubiaceae | *Hedyotis* | 56 | 13.355 | 4.825 | 8.530 | 67.17 | 75.39 | 57.02 | REV+G_RNA16D |  |  |  |  |
| Gentianales | Rubiaceae | *Kadua* | 15 | 11.019 | 4.038 | 6.981 | 61.68 | 71.73 | 47.47 | HKY85+G_RNA16C |  |  |  |  |
| Gentianales | Rubiaceae | *Leptodermis* | 29 | 6.311 | 1.578 | 4.733 | 61.43 | 70.93 | 48.54 | HKY85+G_RNA16D |  |  |  |  |
| Lamiales | Oleaceae | *Fraxinus* | 54 | 11.489 | 5.074 | 6.415 | 64.50 | 76.00 | 46.38 | HKY85+G_RNA16D |  |  |  |  |
| Lamiales | Oleaceae | *Ligustrum* | 19 | 8.533 | 4.067 | 4.466 | 55.90 | 62.97 | 41.62 | REV+G_RNA16D | Premature Convergence | | | |
| Lamiales | Oleaceae | *Olea* | 15 | 25.819 | 9.200 | 16.619 | 67.16 | 71.43 | 61.14 | HKY85+G_RNA16D |  |  |  |  |
| Lamiales | Orobanchaceae | *Triphysaria* | 5 | 12.200 | 2.800 | 9.400 | 61.97 | 72.11 | 44.45 | HKY85+G_RNA7G |  |  |  |  |
| Lamiales | Phrymaceae | *Mimulus* | 89 | 12.447 | 4.877 | 7.570 | 65.87 | 76.51 | 53.48 | REV+G_RNA16D |  |  |  |  |
| Lamiales | Plantaginaceae | *Plantago* | 123 | 19.226 | 8.402 | 10.824 | 54.24 | 66.04 | 37.05 | REV+G_RNA16D |  |  |  |  |
| Laurales | Lauraceae | *Machilus* | 36 | 1.236 | 1.055 | 0.181 | 75.19 | 82.66 | 59.09 | HKY85+G_RNA16C |  |  |  |  |
| Liliales | Smilacaceae | *Smilax* | 109 | 5.897 | 1.704 | 4.193 | 81.39 | 93.12 | 60.28 | REV+G_RNA16A |  |  |  |  |
| Malpighiales | Hypericaceae | *Hypericum* | 103 | 29.289 | 12.708 | 16.581 | 54.38 | 69.57 | 34.13 | REV+G_RNA16D |  |  |  |  |
| Malpighiales | Salicaceae | *Populus* | 44 | 5.544 | 2.027 | 3.517 | 70.46 | 77.55 | 58.37 | REV+G_RNA16I |  |  |  |  |
| Malvales | Malvaceae | *Gossypium* | 45 | 9.042 | 3.496 | 5.546 | 61.84 | 70.52 | 48.60 | HKY85+G_RNA16C |  |  |  |  |
| Myrtales | Onagraceae | *Oenothera* | 76 | 3.679 | 0.965 | 2.714 | 54.43 | 70.53 | 36.09 | HKY85+G_RNA16D |  |  |  |  |
| Piperales | Aristolochiaceae | *Aristolochia* | 46 | 11.265 | 5.650 | 5.614 | 72.91 | 76.76 | 62.86 | HKY85+G_RNA16C |  |  |  |  |
| Poales | Poaceae | *Avena* | 30 | 8.140 | 2.055 | 6.085 | 62.86 | 78.86 | 43.05 | REV+G_RNA16A |  |  |  |  |
| Poales | Poaceae | *Brachypodium* | 16 | 7.850 | 1.833 | 6.017 | 68.50 | 86.08 | 42.28 | HKY85+G_RNA7G |  |  |  |  |
| Poales | Poaceae | *Cenchrus* | 12 | 21.258 | 4.955 | 16.303 | 60.95 | 68.74 | 49.78 | REV+G_RNA16C |  |  |  |  |
| Poales | Poaceae | *Cynodon* | 19 | 4.550 | 1.409 | 3.140 | 57.19 | 60.58 | 50.15 | HKY85+G_RNA16D |  |  |  |  |
| Poales | Poaceae | *Eragrostis* | 127 | 12.292 | 5.225 | 7.067 | 62.02 | 68.04 | 51.43 | REV+G_RNA16D |  |  |  |  |
| Poales | Poaceae | *Leymus* | 35 | 3.965 | 1.531 | 2.434 | 64.56 | 80.33 | 42.76 | REV+G_RNA16A |  |  |  |  |
| Poales | Poaceae | *Lolium* | 15 | 8.571 | 4.210 | 4.361 | 63.88 | 76.45 | 45.03 | REV+G_RNA16C |  |  |  |  |
| Poales | Poaceae | *Panicum* | 53 | 22.967 | 8.942 | 14.025 | 60.62 | 68.85 | 50.31 | REV+G_RNA16D |  |  |  |  |
| Poales | Poaceae | *Phyllostachys* | 15 | 4.857 | 2.629 | 2.228 | 76.65 | 84.19 | 60.47 | HKY85+G_RNA16C |  |  |  |  |
| Poales | Poaceae | *Pseudoroegneria* | 10 | 2.111 | 0.400 | 1.711 | 64.67 | 80.36 | 42.07 | REV+G_RNA7G |  |  |  |  |
| Poales | Poaceae | *Puccinellia* | 37 | 2.529 | 1.724 | 0.805 | 61.86 | 69.97 | 46.59 | HKY85+G_RNA16C |  |  |  |  |
| Poales | Poaceae | *Saccharum* | 11 | 6.709 | 1.273 | 5.436 | 69.89 | 76.77 | 56.51 | HKY85+G_RNA7G |  |  |  |  |
| Poales | Poaceae | *Secale* | 4 | 4.500 | 3.500 | 1.000 | 60.65 | 76.95 | 36.93 | HKY85+G_RNA7E |  |  |  |  |
| Poales | Poaceae | *Triticum* | 14 | 10.297 | 4.143 | 6.154 | 61.91 | 76.13 | 42.17 | REV+G_RNA16A |  |  |  |  |
| Poales | Poaceae | *Zea* | 4 | 2.500 | 0.000 | 2.500 | 74.10 | 86.96 | 52.71 | HKY85+G_RNA7G |  |  |  |  |
| Proteales | Nelumbonaceae | *Nelumbo* | 3 | 2.667 | 1.333 | 1.334 | 57.34 | 62.35 | 47.03 | HKY85+G_RNA7E |  |  |  |  |
| Ranunculales | Berberidaceae | *Dysosma* | 21 | 13.190 | 8.667 | 4.523 | 49.64 | 59.02 | 33.23 | HKY85+G_RNA16D |  |  |  |  |
| Ranunculales | Papaveraceae | *Papaver* | 43 | 20.738 | 5.087 | 15.651 | 58.48 | 65.51 | 50.81 | REV+G_RNA16D |  |  |  |  |
| Ranunculales | Ranunculaceae | *Clematis* | 99 | 7.295 | 2.820 | 4.475 | 67.54 | 77.30 | 51.11 | REV+G_RNA16E |  |  |  |  |
| Rosales | Cannabaceae | *Humulus* | 3 | 16.000 | 6.000 | 10.000 | 57.43 | 70.00 | 38.57 | HKY85+G_RNA7G |  |  |  |  |
| Rosales | Moraceae | *Ficus* | 114 | 11.012 | 5.433 | 5.579 | 68.64 | 80.80 | 49.68 | REV+G_RNA16C | Premature Convergence | | | |
| Rosales | Rosaceae | *Prunus* | 148 | 6.367 | 2.487 | 3.880 | 64.45 | 73.52 | 48.61 | HKY85+G_RNA16D |  |  |  |  |
| Sapindales | Rutaceae | *Citrus* | 49 | 3.330 | 2.043 | 1.287 | 70.52 | 80.57 | 49.30 | REV+G_RNA16J |  |  |  |  |
| Sapindales | Sapindaceae | *Paullinia* | 41 | 18.779 | 4.224 | 14.555 | 66.99 | 79.67 | 53.79 | REV+G_RNA16D |  |  |  |  |
| Solanales | Convolvulaceae | *Cuscuta* | 135 | 39.804 | 19.338 | 20.466 | 51.96 | 64.25 | 36.19 | HKY85+G_RNA16D |  |  |  |  |
| Solanales | Convolvulaceae | *Ipomoea* | 322 | 4.873 | 1.721 | 3.152 | 66.33 | 76.25 | 53.23 | REV+G_RNA16A |  |  |  |  |
| Solanales | Solanaceae | *Capsicum* | 8 | 27.714 | 12.393 | 15.321 | 65.05 | 73.47 | 51.02 | HKY85+G_RNA16A |  |  |  |  |
| Solanales | Solanaceae | *Lycium* | 71 | 9.513 | 5.157 | 4.356 | 69.12 | 81.06 | 51.45 | REV+G_RNA16C |  |  |  |  |
| Solanales | Solanaceae | *Nicotiana* | 69 | 10.508 | 5.449 | 5.059 | 64.45 | 70.96 | 53.93 | HKY85+G_RNA16C |  |  |  |  |
| Solanales | Solanaceae | *Physalis* | 57 | 13.035 | 6.791 | 6.244 | 72.69 | 82.90 | 56.19 | HKY85+G_RNA16C |  |  |  |  |
| Solanales | Solanaceae | *Solanum* | 491 | 3.143 | 1.386 | 1.757 | 73.04 | 81.06 | 63.55 | REV+G_RNA16E |  |  |  |  |
| Vitales | Vitaceae | *Vitis* | 13 | 3.128 | 0.462 | 2.666 | 76.26 | 83.01 | 66.85 | HKY85+G_RNA7G |  |  |  |  |
| Zingiberales | Zingiberaceae | *Zingiber* | 18 | 15.405 | 7.320 | 8.085 | 63.16 | 69.92 | 54.64 | REV+G_RNA16C |  |  |  |  |
| Cycadales | Cycadaceae | *Cycas* | 39 |  | | | 65.11 | 73.48 | 50.99 | HKY85+G_RNA16D |  | | | |
| Cycadales | Zamiaceae | *Picea* | 12 |  |  |  | 58.25 | 64.23 | 51.02 | HKY85+G_RNA7G |  |  |  |  |
| Pinales | Pinaceae | *Pinus* | 72 |  |  |  | 59.84 | 70.72 | 44.39 | REV+G_RNA16D |  |  |  |  |
| Pinales | Pinaceae | *Pseudotsuga* | 5 |  |  |  | 64.48 | 74.92 | 52.08 | HKY85+G_RNA7E |  |  |  |  |
| Pinales | Pinaceae | *Zamia* | 42 |  |  |  | 65.38 | 74.35 | 52.74 | HKY85+G_RNA16C |  |  |  |  |

^a^ GC_p_ and GC_up_ represent GC content in paired and and unpaired regions of ITS2 secondary structure,

Table S2. Comparison of substitution elements from intermediates to GC or AU base-pairs in an initial states of ITS2 rate matrix

| Matrix | Elements of the rate matrix | | | | | | | | | | | | | | | |
| --- | --- | --- | --- | --- | --- | --- | --- | --- | --- | --- | --- | --- | --- | --- | --- | --- |
|  | **GU→GC** | **AC→GC** | **GA→GC** | **UC→GC** | **GG→GC** | **CC→GC** | **NN→GC^a^** | **F(GC)** | **GU→AU** | **AC→AU** | **AG→AU** | **CU→AU** | **AA→AU** | **UU→AU** | **NN→AU^a^** | **F(AU)** |
| *Sagittaria* | 2.1296 | 12.4277 | 1.2005 | 1.8292 | 1.2005 | 1.8292 | 20.6167 | 69.54 | 0.8034 | 7.3481 | 1.0815 | 0.4529 | 1.0815 | 0.4529 | 11.2203 | 15.52 |
| *Allium* | 1.4349 | 4.9305 | 0.7110 | 0.9693 | 0.7110 | 0.9693 | 9.7260 | 45.06 | 1.3083 | 4.6730 | 0.9187 | 0.6483 | 0.9187 | 0.6483 | 9.1153 | 38.94 |
| *Crocus* | 4.2495 | 34.1807 | 6.7431 | 7.8127 | 6.7431 | 7.8127 | 67.5418 | 83.22 | 1.2117 | 13.3289 | 3.0466 | 1.9228 | 3.0466 | 1.9228 | 24.4794 | 9.26 |
| *Aegilops* | 2.8828 | 25.7120 | 8.0661 | 9.8397 | 8.0661 | 9.8397 | 64.4064 | 84.18 | 0.5193 | 8.6003 | 3.2913 | 1.4531 | 3.2913 | 1.4531 | 18.6084 | 5.08 |
| *Agrostis* | 1.6087 | 0.0536 | 0.0536 | 0.0536 | 0.0536 | 0.0536 | 1.8767 | 69.82 | 0.2687 | 0.0090 | 0.0090 | 0.0090 | 0.0090 | 0.0090 | 0.3137 | 9.74 |
| *Eleusine* | 1.6480 | 8.0179 | 3.7887 | 4.9522 | 3.7887 | 4.9522 | 27.1477 | 58.54 | 1.1296 | 6.0422 | 3.7319 | 2.5968 | 3.7319 | 2.5968 | 19.8292 | 30.24 |
| *Hordeum* | 3.3532 | 18.6598 | 1.5871 | 1.9179 | 1.5871 | 1.9179 | 29.0230 | 79.44 | 1.0084 | 7.9101 | 0.8130 | 0.4773 | 0.8130 | 0.4773 | 11.4991 | 10.12 |
| *Oryza* | 1.5020 | 0.2083 | 0.2083 | 0.2083 | 0.2083 | 0.2083 | 2.5435 | 83.45 | 0.1521 | 0.0211 | 0.0211 | 0.0211 | 0.0211 | 0.0211 | 0.2576 | 6.76 |
| *Pennisetum* | 2.6834 | 18.1692 | 0.9995 | 1.4255 | 0.9995 | 1.4255 | 25.7026 | 71.74 | 1.0306 | 10.3825 | 0.8146 | 0.3839 | 0.8146 | 0.3839 | 13.8101 | 15.74 |
| *Setaria* | 1.4453 | 0.0452 | 0.0452 | 0.0452 | 0.0452 | 0.0452 | 1.6713 | 62.56 | 0.7893 | 0.0247 | 0.0247 | 0.0247 | 0.0247 | 0.0247 | 0.9128 | 18.05 |
| *Sorghum* | 3.8187 | 45.9647 | 2.1596 | 2.6776 | 2.1596 | 2.6776 | 59.4578 | 82.06 | 0.9566 | 18.0348 | 1.0506 | 0.5410 | 1.0506 | 0.5410 | 22.1746 | 8.06 |
| *Musa* | 1.3759 | 0.0874 | 0.0874 | 0.0874 | 0.0874 | 0.0874 | 1.8129 | 60.76 | 0.2944 | 0.0187 | 0.0187 | 0.0187 | 0.0187 | 0.0187 | 0.3879 | 15.69 |
| *Curcuma* | 1.8302 | 15.4281 | 0.8959 | 1.2853 | 0.8959 | 1.2853 | 21.6207 | 61.24 | 0.9835 | 10.5611 | 0.8798 | 0.4814 | 0.8798 | 0.4814 | 14.2670 | 22.52 |
| *Eleutherococcus* | 2.3612 | 0.0568 | 0.0568 | 0.0568 | 0.0568 | 0.0568 | 2.6452 | 63.12 | 0.9373 | 0.0226 | 0.0226 | 0.0226 | 0.0226 | 0.0226 | 1.0503 | 21.81 |
| *Hedera* | 2.6615 | 0.1360 | 0.1360 | 0.1360 | 0.1360 | 0.1360 | 3.3415 | 62.96 | 1.2119 | 0.0619 | 0.0619 | 0.0619 | 0.0619 | 0.0619 | 1.5214 | 24.25 |
| *Schefflera* | 2.0944 | 0.1004 | 0.1004 | 0.1004 | 0.1004 | 0.1004 | 2.5964 | 65.32 | 0.7731 | 0.0370 | 0.0370 | 0.0370 | 0.0370 | 0.0370 | 0.9581 | 19.08 |
| *Carthamus* | 1.6705 | 13.1618 | 2.2218 | 3.3847 | 2.2218 | 3.3847 | 26.0453 | 66.00 | 0.7108 | 8.0361 | 2.0666 | 0.9453 | 2.0666 | 0.9453 | 14.7707 | 17.14 |
| *Helianthus* | 1.9743 | 21.3677 | 3.7200 | 5.4638 | 3.7200 | 5.4638 | 41.7096 | 66.96 | 0.8631 | 13.3357 | 3.4100 | 1.6263 | 3.4100 | 1.6263 | 24.2714 | 18.28 |
| *Lactuca* | 1.8179 | 10.8955 | 1.0423 | 1.4731 | 1.0423 | 1.4731 | 17.7442 | 65.04 | 0.8242 | 6.7913 | 0.9182 | 0.4726 | 0.9182 | 0.4726 | 10.3971 | 18.38 |
| *Gaillardia* | 0.7949 | 0.0687 | 0.0687 | 0.0687 | 0.0687 | 0.0687 | 1.1384 | 47.25 | 0.9011 | 0.0779 | 0.0779 | 0.0779 | 0.0779 | 0.0779 | 1.2906 | 26.87 |
| *Lasthenia* | 0.8291 | 0.0498 | 0.0498 | 0.0498 | 0.0498 | 0.0498 | 1.0781 | 49.07 | 0.8904 | 0.0535 | 0.0535 | 0.0535 | 0.0535 | 0.0535 | 1.1579 | 28.84 |
| *Sinosenecio* | 1.2373 | 0.0706 | 0.0706 | 0.0706 | 0.0706 | 0.0706 | 1.5903 | 54.07 | 0.6647 | 0.0379 | 0.0379 | 0.0379 | 0.0379 | 0.0379 | 0.8542 | 24.24 |
| *Arabidopsis* | 2.4599 | 10.3255 | 2.3584 | 2.8215 | 2.3584 | 2.8215 | 23.1452 | 69.68 | 1.2050 | 5.9226 | 1.6184 | 1.1552 | 1.6184 | 1.1552 | 12.6748 | 19.58 |
| *Noccaea* | 2.8656 | 25.5715 | 3.6143 | 4.8104 | 3.6143 | 4.8104 | 45.2865 | 65.52 | 1.6131 | 17.2294 | 3.2411 | 2.0346 | 3.2411 | 2.0346 | 29.3939 | 24.84 |
| *Cucumis* | 8.4178 | 26.9095 | 5.4198 | 5.8333 | 5.4198 | 5.8333 | 57.8335 | 84.90 | 2.9174 | 10.2519 | 2.2223 | 1.8783 | 2.2223 | 1.8783 | 21.3705 | 11.22 |
| *Rhododendron* | 3.4418 | 34.7261 | 1.5807 | 1.9550 | 1.5807 | 1.9550 | 45.2393 | 71.74 | 1.6113 | 19.6811 | 1.1080 | 0.7400 | 1.1080 | 0.7400 | 24.9884 | 19.04 |
| *Cyclamen* | 1.9143 | 0.0692 | 0.0692 | 0.0692 | 0.0692 | 0.0692 | 2.2603 | 71.20 | 0.7324 | 0.0265 | 0.0265 | 0.0265 | 0.0265 | 0.0265 | 0.8649 | 17.71 |
| *Arachis* | 3.6856 | 18.1141 | 5.4185 | 6.3611 | 5.4185 | 6.3611 | 45.3589 | 86.00 | 0.7996 | 5.9818 | 2.1006 | 1.1756 | 2.1006 | 1.1756 | 13.3338 | 6.16 |
| *Cicer* | 2.0554 | 22.8742 | 4.6753 | 6.8172 | 4.6753 | 6.8172 | 47.9146 | 56.28 | 1.4473 | 18.3991 | 5.4834 | 3.2921 | 5.4834 | 3.2921 | 37.3974 | 31.88 |
| *Cyamopsis* | 1.3178 | 5.9982 | 1.8646 | 2.7107 | 1.8646 | 2.7107 | 16.4666 | 52.02 | 0.9966 | 5.0621 | 2.2877 | 1.4102 | 2.2877 | 1.4102 | 13.4545 | 33.20 |
| *Glycine* | 2.5163 | 12.7787 | 1.4103 | 1.8360 | 1.4103 | 1.8360 | 21.7876 | 66.44 | 1.3212 | 8.0886 | 1.1622 | 0.7405 | 1.1622 | 0.7405 | 13.2152 | 22.08 |
| *Salvia* | 1.8765 | 0.1327 | 0.1327 | 0.1327 | 0.1327 | 0.1327 | 2.5400 | 73.43 | 1.8765 | 0.1327 | 0.1327 | 0.1327 | 0.1327 | 0.1327 | 2.5400 | 13.99 |
| *Antirrhinum* | 3.6230 | 26.4956 | 3.3322 | 4.0426 | 3.3322 | 4.0426 | 44.8682 | 77.52 | 1.3149 | 12.8699 | 1.9637 | 1.2094 | 1.9637 | 1.2094 | 20.5310 | 13.66 |
| *Persea* | 4.9629 | 56.9385 | 2.3786 | 2.7010 | 2.3786 | 2.7010 | 72.0606 | 88.96 | 0.6123 | 12.9312 | 0.6134 | 0.2935 | 0.6134 | 0.2935 | 15.3573 | 2.50 |
| *Euphorbia* | 1.9925 | 0.1026 | 0.1026 | 0.1026 | 0.1026 | 0.1026 | 2.5055 | 70.46 | 0.3852 | 0.0198 | 0.0198 | 0.0198 | 0.0198 | 0.0198 | 0.4842 | 12.56 |
| *Asarum* | 2.5133 | 19.8792 | 0.4187 | 0.5893 | 0.4187 | 0.5893 | 24.4085 | 62.58 | 1.4501 | 13.9840 | 0.4146 | 0.2416 | 0.4146 | 0.2416 | 16.7465 | 25.40 |
| *Eschscholzia* | 1.4587 | 0.1871 | 0.1871 | 0.1871 | 0.1871 | 0.1871 | 2.3942 | 78.96 | 0.1417 | 0.0182 | 0.0182 | 0.0182 | 0.0182 | 0.0182 | 0.2327 | 9.89 |
| *Adonis* | 4.1417 | 23.8544 | 4.7176 | 5.8943 | 4.7176 | 5.8943 | 49.2199 | 65.34 | 2.5777 | 16.7338 | 4.1348 | 2.9362 | 4.1348 | 2.9362 | 33.4535 | 28.54 |
| *Fragaria* | 3.7348 | 26.3527 | 9.0543 | 11.2364 | 9.0543 | 11.2364 | 70.6689 | 82.78 | 1.0375 | 11.1903 | 4.7714 | 2.5152 | 4.7714 | 2.5152 | 26.8010 | 9.76 |
| *Malus* | 1.5112 | 6.4202 | 3.2118 | 3.8871 | 3.2118 | 3.8871 | 22.1292 | 77.76 | 0.3792 | 2.5076 | 1.5182 | 0.8059 | 1.5182 | 0.8059 | 7.5350 | 7.62 |
| *Rosa* | 3.8675 | 81.5369 | 5.4751 | 7.0367 | 5.4751 | 7.0367 | 110.4280 | 74.68 | 1.5889 | 44.2080 | 3.8152 | 2.2494 | 3.8152 | 2.2494 | 57.9261 | 16.64 |
| *Cedrela* | 1.3486 | 0.2330 | 0.2330 | 0.2330 | 0.2330 | 0.2330 | 2.5136 | 79.12 | 0.4237 | 0.0732 | 0.0732 | 0.0732 | 0.0732 | 0.0732 | 0.7897 | 13.21 |
| *Sedum* | 1.1962 | 0.1342 | 0.1342 | 0.1342 | 0.1342 | 0.1342 | 1.8672 | 53.75 | 0.5002 | 0.0561 | 0.0561 | 0.0561 | 0.0561 | 0.0561 | 0.7807 | 22.44 |
| *Ribes* | 2.9548 | 21.9287 | 3.6120 | 4.6598 | 3.6120 | 4.6598 | 41.4271 | 71.36 | 1.3420 | 12.7812 | 2.7160 | 1.6405 | 2.7160 | 1.6405 | 22.8362 | 18.88 |
| *Paeonia* | 1.5078 | 0.1549 | 0.1549 | 0.1549 | 0.1549 | 0.1549 | 2.2823 | 58.77 | 1.3192 | 0.1355 | 0.1355 | 0.1355 | 0.1355 | 0.1355 | 1.9967 | 24.63 |
| *Petunia* | 1.5085 | 0.4192 | 0.4192 | 0.4192 | 0.4192 | 0.4192 | 3.6045 | 61.04 | 0.6414 | 0.1782 | 0.1782 | 0.1782 | 0.1782 | 0.1782 | 1.5324 | 22.63 |
| *Acorus* | 2.0356 | 12.3146 | 6.7501 | 7.9165 | 6.7501 | 7.9165 | 43.6834 | 85.60 | 0.2711 | 3.2721 | 2.1035 | 0.8988 | 2.1035 | 0.8988 | 9.5478 | 3.02 |
| *Asparagus* | 1.9891 | 0.1113 | 0.1113 | 0.1113 | 0.1113 | 0.1113 | 2.5456 | 67.58 | 0.3226 | 0.0180 | 0.0180 | 0.0180 | 0.0180 | 0.0180 | 0.4126 | 12.37 |
| *Iris* | 2.8809 | 3.1585 | 0.5412 | 0.5412 | 0.5412 | 0.5412 | 8.2042 | 71.40 | 0.5276 | 0.4812 | 0.0904 | 0.0904 | 0.0904 | 0.0904 | 1.3704 | 13.29 |
| *Phalaenopsis* | 2.7253 | 15.5669 | 1.2803 | 1.6004 | 1.2803 | 1.6004 | 24.0536 | 80.10 | 0.5653 | 5.6867 | 0.5846 | 0.2656 | 0.5846 | 0.2656 | 7.9524 | 6.06 |
| *Habenaria* | 1.9724 | 16.2127 | 1.6276 | 2.3177 | 1.6276 | 2.3177 | 26.0757 | 46.40 | 1.8763 | 15.4986 | 2.2156 | 1.5483 | 2.2156 | 1.5483 | 24.9027 | 42.18 |
| *Holcoglossum* | 4.5105 | 33.5421 | 7.3803 | 8.3855 | 7.3803 | 8.3855 | 69.5842 | 85.28 | 1.1414 | 11.8838 | 2.9709 | 1.8675 | 2.9709 | 1.8675 | 22.7020 | 7.64 |
| *Smilax* | 6.9799 | 0.7519 | 0.7519 | 0.7519 | 0.7519 | 0.7519 | 10.7394 | 90.08 | 0.2216 | 0.0239 | 0.0239 | 0.0239 | 0.0239 | 0.0239 | 0.3411 | 3.84 |
| *Avena* | 3.1780 | 0.2100 | 0.2100 | 0.2100 | 0.2100 | 0.2100 | 4.2280 | 73.34 | 0.1467 | 0.0097 | 0.0097 | 0.0097 | 0.0097 | 0.0097 | 0.1952 | 14.71 |
| *Cenchrus* | 1.1042 | 0.0664 | 0.0664 | 0.0664 | 0.0664 | 0.0664 | 1.4362 | 59.70 | 0.9961 | 0.0599 | 0.0599 | 0.0599 | 0.0599 | 0.0599 | 1.2956 | 20.60 |
| *Cynodon* | 4.0109 | 29.5966 | 8.7107 | 11.4233 | 8.7107 | 11.4233 | 73.8755 | 66.52 | 2.3925 | 20.4901 | 7.9084 | 5.1959 | 7.9084 | 5.1959 | 49.0912 | 27.48 |
| *Eragrostis* | 3.7135 | 12.6638 | 2.0134 | 2.5051 | 2.0134 | 2.5051 | 25.4143 | 75.48 | 1.5358 | 6.5181 | 1.2894 | 0.8327 | 1.2894 | 0.8327 | 12.2981 | 16.06 |
| *Panicum* | 3.4172 | 12.0909 | 1.0494 | 1.3362 | 1.0494 | 1.3362 | 20.2793 | 75.16 | 1.2890 | 6.1868 | 0.6837 | 0.3959 | 0.6837 | 0.3959 | 9.6350 | 14.50 |
| *Leymus* | 2.3535 | 0.1410 | 0.1410 | 0.1410 | 0.1410 | 0.1410 | 3.0585 | 72.04 | 0.4538 | 0.0272 | 0.0272 | 0.0272 | 0.0272 | 0.0272 | 0.5898 | 11.39 |
| *Lolium* | 1.8414 | 0.1059 | 0.1059 | 0.1059 | 0.1059 | 0.1059 | 2.3709 | 68.73 | 0.4839 | 0.0278 | 0.0278 | 0.0278 | 0.0278 | 0.0278 | 0.6229 | 15.83 |
| *Phyllostachys* | 3.7203 | 0.4212 | 0.4212 | 0.4212 | 0.4212 | 0.4212 | 5.8263 | 79.74 | 0.8608 | 0.0975 | 0.0975 | 0.0975 | 0.0975 | 0.0975 | 1.3483 | 11.37 |
| *Puccinellia* | 2.8167 | 0.2685 | 0.2685 | 0.2685 | 0.2685 | 0.2685 | 4.1592 | 63.55 | 1.0595 | 0.1010 | 0.1010 | 0.1010 | 0.1010 | 0.1010 | 1.5645 | 23.61 |
| *Triticum* | 2.4997 | 0.1026 | 0.1026 | 0.1026 | 0.1026 | 0.1026 | 3.0127 | 68.59 | 0.4947 | 0.0203 | 0.0203 | 0.0203 | 0.0203 | 0.0203 | 0.5962 | 16.33 |
| *Zingiber* | 2.2696 | 0.1079 | 0.1079 | 0.1079 | 0.1079 | 0.1079 | 2.8091 | 61.90 | 0.5663 | 0.0269 | 0.0269 | 0.0269 | 0.0269 | 0.0269 | 0.7008 | 22.05 |
| *Apium* | 1.2870 | 0.0996 | 0.0996 | 0.0996 | 0.0996 | 0.0996 | 1.7850 | 55.36 | 0.9837 | 0.0761 | 0.0761 | 0.0761 | 0.0761 | 0.0761 | 1.3642 | 25.80 |
| *Angelica* | 3.3584 | 11.2428 | 3.0562 | 3.5561 | 3.0562 | 3.5561 | 27.8258 | 77.10 | 1.3294 | 5.3846 | 1.7031 | 1.2098 | 1.7031 | 1.2098 | 12.5398 | 14.62 |
| *Bupleurum* | 1.5920 | 0.1337 | 0.1337 | 0.1337 | 0.1337 | 0.1337 | 2.2605 | 62.02 | 0.6815 | 0.0572 | 0.0572 | 0.0572 | 0.0572 | 0.0572 | 0.9675 | 20.79 |
| *Aralia* | 3.6342 | 15.4109 | 3.3471 | 3.6673 | 3.3471 | 3.6673 | 33.0739 | 81.38 | 1.1720 | 6.0464 | 1.4389 | 1.0794 | 1.4389 | 1.0794 | 12.2550 | 10.30 |
| *Dendropanax* | 2.6160 | 9.4796 | 1.3970 | 1.5784 | 1.3970 | 1.5784 | 18.0464 | 76.72 | 0.8967 | 3.8995 | 0.6493 | 0.4789 | 0.6493 | 0.4789 | 7.0526 | 10.82 |
| *Panax* | 1.9735 | 0.1887 | 0.1887 | 0.1887 | 0.1887 | 0.1887 | 2.9170 | 65.95 | 0.6925 | 0.0662 | 0.0662 | 0.0662 | 0.0662 | 0.0662 | 1.0235 | 21.29 |
| *Macropanax* | 2.0278 | 0.2419 | 0.2419 | 0.2419 | 0.2419 | 0.2419 | 3.2373 | 65.33 | 0.7480 | 0.0892 | 0.0892 | 0.0892 | 0.0892 | 0.0892 | 1.1940 | 18.84 |
| *Oplopanax* | 1.1960 | 0.3544 | 0.3544 | 0.3544 | 0.3544 | 0.3544 | 2.9680 | 66.20 | 0.6124 | 0.1814 | 0.1814 | 0.1814 | 0.1814 | 0.1814 | 1.5194 | 21.30 |
| *Barnadesia* | 3.6574 | 15.9530 | 2.5897 | 3.1731 | 2.5897 | 3.1731 | 31.1360 | 71.74 | 1.7783 | 9.3367 | 1.8571 | 1.2592 | 1.8571 | 1.2592 | 17.3476 | 20.42 |
| *Cynara* | 0.9679 | 0.2007 | 0.2007 | 0.2007 | 0.2007 | 0.2007 | 1.9714 | 58.36 | 0.4482 | 0.0930 | 0.0930 | 0.0930 | 0.0930 | 0.0930 | 0.9132 | 19.30 |
| *Stevia* | 1.1727 | 8.6587 | 0.8721 | 1.3604 | 0.8721 | 1.3604 | 14.2964 | 52.74 | 0.7143 | 6.6406 | 1.0433 | 0.5312 | 1.0433 | 0.5312 | 10.5039 | 24.64 |
| *Chaetanthera* | 1.6067 | 6.3895 | 0.8469 | 1.1215 | 0.8469 | 1.1215 | 11.9330 | 71.64 | 0.3816 | 2.6642 | 0.4676 | 0.2011 | 0.4676 | 0.2011 | 4.3832 | 7.10 |
| *Chrysanthemum* | 2.7386 | 25.7730 | 1.2140 | 1.5483 | 1.2140 | 1.5483 | 34.0362 | 68.92 | 1.3195 | 15.2419 | 0.9157 | 0.5849 | 0.9157 | 0.5849 | 19.5626 | 19.64 |
| *Dahlia* | 2.0488 | 11.8625 | 1.3473 | 1.8196 | 1.3473 | 1.8196 | 20.2451 | 68.46 | 0.8586 | 6.9014 | 1.0586 | 0.5646 | 1.0586 | 0.5646 | 11.0064 | 16.68 |
| *Flaveria* | 1.2196 | 6.6828 | 0.5005 | 0.7933 | 0.5005 | 0.7933 | 10.4900 | 49.56 | 0.8578 | 5.5728 | 0.6615 | 0.3520 | 0.6615 | 0.3520 | 8.4576 | 29.06 |
| *Lessingia* | 2.1714 | 33.2911 | 2.1713 | 3.0323 | 2.1713 | 3.0323 | 45.8697 | 54.06 | 1.6435 | 27.4582 | 2.5010 | 1.6435 | 2.5010 | 1.6435 | 37.3907 | 33.76 |
| *Melampodium* | 1.6728 | 6.1798 | 0.7483 | 1.0047 | 0.7483 | 1.0047 | 11.3586 | 64.06 | 0.7478 | 3.8225 | 0.6214 | 0.3345 | 0.6214 | 0.3345 | 6.4821 | 17.72 |
| *Senecio* | 0.8806 | 0.0293 | 0.0293 | 0.0293 | 0.0293 | 0.0293 | 1.0271 | 50.11 | 0.6210 | 0.0207 | 0.0207 | 0.0207 | 0.0207 | 0.0207 | 0.7245 | 27.14 |
| *Taraxacum* | 1.0805 | 0.0460 | 0.0460 | 0.0460 | 0.0460 | 0.0460 | 1.3105 | 53.05 | 0.7797 | 0.0332 | 0.0332 | 0.0332 | 0.0332 | 0.0332 | 0.9457 | 24.18 |
| *Zinnia* | 0.7907 | 0.0507 | 0.0507 | 0.0507 | 0.0507 | 0.0507 | 1.0442 | 48.96 | 0.8023 | 0.0515 | 0.0515 | 0.0515 | 0.0515 | 0.0515 | 1.0598 | 27.81 |
| *Brassica* | 1.3186 | 0.0636 | 0.0636 | 0.0636 | 0.0636 | 0.0636 | 1.6366 | 58.02 | 0.4804 | 0.0232 | 0.0232 | 0.0232 | 0.0232 | 0.0232 | 0.5964 | 21.35 |
| *Draba* | 2.5115 | 22.1663 | 5.1125 | 6.3847 | 5.1125 | 6.3847 | 47.6722 | 72.70 | 1.0457 | 11.7037 | 3.3711 | 2.1286 | 3.3711 | 2.1286 | 23.7488 | 15.98 |
| *Mesembryanthemum* | 1.7319 | 0.4367 | 0.4367 | 0.4367 | 0.4367 | 0.4367 | 3.9154 | 78.62 | 0.2979 | 0.0751 | 0.0751 | 0.0751 | 0.0751 | 0.0751 | 0.6734 | 8.48 |
| *Silene* | 2.1497 | 0.0714 | 0.0714 | 0.0714 | 0.0714 | 0.0714 | 2.5067 | 64.43 | 0.5863 | 0.0195 | 0.0195 | 0.0195 | 0.0195 | 0.0195 | 0.6838 | 19.82 |
| *Limonium* | 1.0273 | 6.1733 | 0.6749 | 1.1370 | 0.6749 | 1.1370 | 10.8244 | 46.42 | 0.7794 | 5.3668 | 0.9884 | 0.5120 | 0.9884 | 0.5120 | 9.1470 | 30.60 |
| *Persicaria* | 3.3743 | 10.5785 | 2.0511 | 2.2860 | 2.0511 | 2.2860 | 22.6270 | 82.72 | 0.8905 | 3.5280 | 0.7624 | 0.5413 | 0.7624 | 0.5413 | 7.0259 | 7.28 |
| *Tamarix* | 2.5343 | 0.1293 | 0.1293 | 0.1293 | 0.1293 | 0.1293 | 3.1808 | 68.99 | 0.8448 | 0.0431 | 0.0431 | 0.0431 | 0.0431 | 0.0431 | 1.0603 | 15.79 |
| *Euonymus* | 3.2305 | 0.2189 | 0.2189 | 0.2189 | 0.2189 | 0.2189 | 4.3250 | 77.08 | 0.9100 | 0.0617 | 0.0617 | 0.0617 | 0.0617 | 0.0617 | 1.2185 | 13.09 |
| *Parnassia* | 1.7505 | 4.9195 | 1.4554 | 1.8722 | 1.4554 | 1.8722 | 13.3252 | 57.98 | 1.1547 | 3.7035 | 1.4094 | 0.9601 | 1.4094 | 0.9601 | 9.5972 | 28.80 |
| *Hemsleya* | 4.3235 | 1.3545 | 1.3545 | 1.3545 | 1.3545 | 1.3545 | 11.0960 | 71.24 | 1.2987 | 0.4069 | 0.4069 | 0.4069 | 0.4069 | 0.4069 | 3.3332 | 21.58 |
| *Viburnum* | 1.2685 | 0.0842 | 0.0842 | 0.0842 | 0.0842 | 0.0842 | 1.6895 | 66.64 | 0.5383 | 0.0357 | 0.0357 | 0.0357 | 0.0357 | 0.0357 | 0.7168 | 18.21 |
| *Actinidia* | 2.8115 | 17.4396 | 1.0880 | 1.3868 | 1.0880 | 1.3868 | 25.2007 | 61.82 | 1.8063 | 12.3968 | 0.9858 | 0.6990 | 0.9858 | 0.6990 | 17.5727 | 28.22 |
| *Diospyros* | 4.1441 | 19.2183 | 3.3530 | 3.7430 | 3.3530 | 3.7430 | 37.5544 | 89.58 | 0.4282 | 3.5719 | 0.6957 | 0.3465 | 0.6957 | 0.3465 | 6.0845 | 1.72 |
| *Vaccinium* | 3.6146 | 20.6926 | 0.9130 | 1.0955 | 0.9130 | 1.0955 | 28.3242 | 80.06 | 1.0656 | 8.5181 | 0.4510 | 0.2692 | 0.4510 | 0.2692 | 11.0241 | 9.72 |
| *Enkianthus* | 1.6350 | 0.0924 | 0.0924 | 0.0924 | 0.0924 | 0.0924 | 2.0970 | 65.52 | 1.0148 | 0.0573 | 0.0573 | 0.0573 | 0.0573 | 0.0573 | 1.3013 | 19.28 |
| *Lysimachia* | 1.6131 | 0.0574 | 0.0574 | 0.0574 | 0.0574 | 0.0574 | 1.9001 | 64.28 | 0.4859 | 0.0173 | 0.0173 | 0.0173 | 0.0173 | 0.0173 | 0.5724 | 14.65 |
| *Lathyrus* | 1.5225 | 8.8738 | 2.3929 | 3.4496 | 2.3929 | 3.4496 | 22.0813 | 65.04 | 1.1164 | 7.2791 | 2.8297 | 1.7547 | 2.8297 | 1.7547 | 17.5643 | 18.38 |
| *Lotus* | 1.9127 | 7.3603 | 1.0069 | 1.4150 | 1.0069 | 1.4150 | 14.1168 | 60.48 | 1.1471 | 5.1424 | 0.9886 | 0.6039 | 0.9886 | 0.6039 | 9.4745 | 25.34 |
| *Lupinus* | 2.3948 | 0.5242 | 0.5242 | 0.5242 | 0.5242 | 0.5242 | 5.0158 | 59.44 | 0.5363 | 0.1174 | 0.1174 | 0.1174 | 0.1174 | 0.1174 | 1.1233 | 20.08 |
| *Medicago* | 2.6616 | 17.3580 | 2.7824 | 3.6953 | 2.7824 | 3.6953 | 32.9750 | 45.41 | 1.9088 | 13.9060 | 2.9604 | 1.9955 | 2.9604 | 1.9955 | 25.7266 | 33.14 |
| *Trifolium* | 1.7748 | 10.3510 | 1.3910 | 2.0386 | 1.3910 | 2.0386 | 18.9850 | 54.78 | 1.2425 | 8.4510 | 1.6644 | 0.9738 | 1.6644 | 0.9738 | 14.9699 | 31.32 |
| *Vicia* | 1.4656 | 13.7777 | 1.4746 | 2.3086 | 1.4746 | 2.3086 | 22.8097 | 53.54 | 1.0092 | 10.8412 | 1.8165 | 1.0153 | 1.8165 | 1.0153 | 17.5140 | 29.00 |
| *Phaseolus* | 4.3515 | 21.7990 | 1.2085 | 1.4483 | 1.2085 | 1.4483 | 31.4641 | 71.32 | 2.2049 | 12.9359 | 0.8595 | 0.6124 | 0.8595 | 0.6124 | 18.0846 | 21.44 |
| *Vigna* | 3.0841 | 14.9333 | 1.2685 | 1.6300 | 1.2685 | 1.6300 | 23.8144 | 57.74 | 2.2796 | 11.7895 | 1.2869 | 0.9376 | 1.2869 | 0.9376 | 18.5181 | 33.70 |
| *Astragalus* | 2.1619 | 10.1472 | 1.2168 | 1.5696 | 1.2168 | 1.5696 | 17.8819 | 61.84 | 1.2929 | 6.9981 | 1.0825 | 0.7277 | 1.0825 | 0.7277 | 11.9114 | 25.50 |
| *Betula* | 2.1239 | 0.1116 | 0.1116 | 0.1116 | 0.1116 | 0.1116 | 2.6819 | 68.26 | 0.6115 | 0.0321 | 0.0321 | 0.0321 | 0.0321 | 0.0321 | 0.7720 | 17.41 |
| *Quercus* | 3.0257 | 20.6554 | 1.3750 | 1.6367 | 1.3750 | 1.6367 | 29.7045 | 77.54 | 0.9754 | 9.0852 | 0.7199 | 0.4433 | 0.7199 | 0.4433 | 12.3870 | 11.00 |
| *Juglans* | 2.8140 | 0.5731 | 0.5731 | 0.5731 | 0.5731 | 0.5731 | 5.6795 | 67.84 | 1.1558 | 0.2354 | 0.2354 | 0.2354 | 0.2354 | 0.2354 | 2.3328 | 20.63 |
| *Swertia* | 2.5578 | 9.1315 | 1.3498 | 1.6230 | 1.3498 | 1.6230 | 17.6349 | 75.36 | 0.8741 | 4.2175 | 0.7496 | 0.4613 | 0.7496 | 0.4613 | 7.5134 | 11.90 |
| *Coffea* | 2.8401 | 0.2271 | 0.2271 | 0.2271 | 0.2271 | 0.2271 | 3.9756 | 70.07 | 1.0862 | 0.0868 | 0.0868 | 0.0868 | 0.0868 | 0.0868 | 1.5202 | 16.66 |
| *Hedyotis* | 4.1489 | 16.2954 | 2.1438 | 2.5396 | 2.1438 | 2.5396 | 29.8111 | 83.14 | 1.0981 | 6.1680 | 0.9613 | 0.5674 | 0.9613 | 0.5674 | 10.3235 | 8.32 |
| *Kadua* | 2.1157 | 0.1037 | 0.1037 | 0.1037 | 0.1037 | 0.1037 | 2.6342 | 64.02 | 0.5865 | 0.0288 | 0.0288 | 0.0288 | 0.0288 | 0.0288 | 0.7305 | 19.34 |
| *Leptodermis* | 8.2996 | 64.9807 | 5.9506 | 6.4611 | 5.9506 | 6.4611 | 98.1037 | 82.34 | 3.1917 | 28.1494 | 2.7989 | 2.2884 | 2.7989 | 2.2884 | 41.5157 | 13.72 |
| *Fraxinus* | 2.4899 | 21.3330 | 2.0777 | 2.5781 | 2.0777 | 2.5781 | 33.1345 | 78.52 | 0.5637 | 8.2741 | 0.9999 | 0.4703 | 0.9999 | 0.4703 | 11.7782 | 6.90 |
| *Olea* | 2.5584 | 13.4847 | 2.1035 | 2.5157 | 2.1035 | 2.5157 | 25.2815 | 76.18 | 0.8934 | 5.9653 | 1.1129 | 0.7345 | 1.1129 | 0.7345 | 10.5535 | 11.76 |
| *Ligustrum* | 3.0273 | 25.0503 | 2.6667 | 3.6085 | 2.6667 | 3.6085 | 40.6280 | 67.96 | 1.5430 | 16.1790 | 2.3306 | 1.3592 | 2.3306 | 1.3592 | 25.1016 | 22.38 |
| *Mimulus* | 3.1029 | 15.6758 | 2.7624 | 3.2105 | 2.7624 | 3.2105 | 30.7245 | 82.20 | 0.7460 | 5.7099 | 1.1694 | 0.6641 | 1.1694 | 0.6641 | 10.1229 | 7.20 |
| *Plantago* | 2.2513 | 7.0539 | 2.3432 | 2.8408 | 2.3432 | 2.8408 | 19.6732 | 70.94 | 0.9807 | 4.0844 | 1.6449 | 1.0207 | 1.6449 | 1.0207 | 10.3963 | 17.90 |
| *Machilus* | 2.8798 | 3.4951 | 3.4951 | 3.4951 | 3.4951 | 3.4951 | 20.3553 | 76.86 | 0.6805 | 0.8259 | 0.8259 | 0.8259 | 0.8259 | 0.8259 | 4.8100 | 11.55 |
| *Hypericum* | 1.4963 | 4.9838 | 1.5926 | 1.8803 | 1.5926 | 1.8803 | 13.4259 | 70.12 | 0.5547 | 2.4613 | 0.9286 | 0.5904 | 0.9286 | 0.5904 | 6.0540 | 12.84 |
| *Populus* | 3.9271 | 0.6082 | 0.3798 | 0.0630 | 1.0366 | 1.0366 | 7.0513 | 71.61 | 0.1630 | 1.0528 | 0.0169 | 0.1018 | 0.0318 | 0.0318 | 1.3981 | 16.50 |
| *Gossypium* | 1.7740 | 0.1636 | 0.1636 | 0.1636 | 0.1636 | 0.1636 | 2.5920 | 63.26 | 0.7755 | 0.0715 | 0.0715 | 0.0715 | 0.0715 | 0.0715 | 1.1330 | 22.22 |
| *Oenothera* | 3.4096 | 30.4281 | 11.5402 | 13.7547 | 11.5402 | 13.7547 | 84.4275 | 78.82 | 1.2206 | 14.2408 | 6.4374 | 4.1314 | 6.4374 | 4.1314 | 36.5990 | 13.20 |
| *Aristolochia* | 1.5589 | 0.1077 | 0.1077 | 0.1077 | 0.1077 | 0.1077 | 2.0974 | 68.63 | 0.4820 | 0.0333 | 0.0333 | 0.0333 | 0.0333 | 0.0333 | 0.6485 | 13.54 |
| *Dysosma* | 3.2726 | 10.2214 | 0.8353 | 0.9819 | 0.8353 | 0.9819 | 17.1284 | 61.44 | 2.1276 | 7.4803 | 0.7186 | 0.5431 | 0.7186 | 0.5431 | 12.1313 | 29.24 |
| *Papaver* | 3.3373 | 16.4132 | 2.1518 | 2.6413 | 2.1518 | 2.6413 | 29.3367 | 71.78 | 1.5795 | 9.3542 | 1.5054 | 1.0184 | 1.5054 | 1.0184 | 15.9813 | 19.36 |
| *Clematis* | 16.5752 | 17.1810 | 2.3449 | 2.4307 | 2.3449 | 2.4307 | 43.3074 | 87.40 | 4.6101 | 5.3043 | 0.7504 | 0.6522 | 0.7504 | 0.6522 | 12.7196 | 7.50 |
| *Ficus* | 1.9837 | 0.1318 | 0.1318 | 0.1318 | 0.1318 | 0.1318 | 2.6427 | 74.00 | 0.4564 | 0.0303 | 0.0303 | 0.0303 | 0.0303 | 0.0303 | 0.6079 | 12.39 |
| *Prunus* | 2.7031 | 20.4287 | 2.3918 | 2.9412 | 2.3918 | 2.9412 | 33.7978 | 78.10 | 0.7752 | 8.5750 | 1.2346 | 0.6859 | 1.2346 | 0.6859 | 13.1912 | 9.40 |
| *Citrus* | 3.3649 | 2.8536 | 1.8505 | 1.8505 | 1.8505 | 1.8505 | 13.6205 | 75.99 | 0.7707 | 0.9088 | 0.4998 | 0.4998 | 0.4998 | 0.4998 | 3.6787 | 13.74 |
| *Paullinia* | 3.1101 | 17.3119 | 2.0086 | 2.3848 | 2.0086 | 2.3848 | 29.2088 | 83.34 | 0.5987 | 5.3293 | 0.7341 | 0.3867 | 0.7341 | 0.3867 | 8.1696 | 4.94 |
| *Cuscuta* | 0.9545 | 2.8862 | 0.6368 | 0.9501 | 0.6368 | 0.9501 | 7.0145 | 57.06 | 0.4258 | 1.8195 | 0.5989 | 0.2841 | 0.5989 | 0.2841 | 4.0113 | 16.04 |
| *Ipomoea* | 1.3616 | 0.0869 | 0.0869 | 0.0869 | 0.0869 | 0.0869 | 1.7961 | 65.54 | 0.4374 | 0.0279 | 0.0279 | 0.0279 | 0.0279 | 0.0279 | 0.5769 | 13.04 |
| *Capsicum* | 1.8543 | 0.2380 | 0.2380 | 0.2380 | 0.2380 | 0.2380 | 3.0443 | 66.22 | 0.4905 | 0.0630 | 0.0630 | 0.0630 | 0.0630 | 0.0630 | 0.8055 | 14.31 |
| *Solanum* | 6.3138 | 7.0844 | 1.2589 | 1.4125 | 1.2589 | 1.4125 | 18.7410 | 84.16 | 1.1764 | 1.9538 | 0.3896 | 0.2346 | 0.3896 | 0.2346 | 4.3786 | 4.32 |
| *Nicotiana* | 1.8351 | 0.1039 | 0.1039 | 0.1039 | 0.1039 | 0.1039 | 2.3546 | 62.64 | 1.0349 | 0.0586 | 0.0586 | 0.0586 | 0.0586 | 0.0586 | 1.3279 | 20.71 |
| *Lycium* | 2.1810 | 0.1357 | 0.1357 | 0.1357 | 0.1357 | 0.1357 | 2.8595 | 75.21 | 0.4002 | 0.0249 | 0.0249 | 0.0249 | 0.0249 | 0.0249 | 0.5247 | 13.09 |
| *Physalis* | 1.8614 | 0.1711 | 0.1711 | 0.1711 | 0.1711 | 0.1711 | 2.7169 | 77.64 | 0.4384 | 0.0403 | 0.0403 | 0.0403 | 0.0403 | 0.0403 | 0.6399 | 11.84 |
| Average | 2.6160 | 10.4237 | 1.6260 | 1.9950 | 1.6307 | 2.0019 | 20.2932 | 68.69 | 0.9832 | 5.4422 | 1.0205 | 0.6435 | 1.0206 | 0.6430 | 9.7530 | 17.63 |

^a^ NN→GC/AU represent the sum substitution rates from the six intermediates to GC or AU; F_(GC)_/ F_(AU)_ represent the GC or AU frequency in ITS2 rate matrix

Table S3. Comparison of substitution elements from intermediates to GC or AU base-pairs at equilibrium states of ITS2 rate matrix

| Matrix | Elements of the rate matrix | | | | | | | | | | | | | | | |
| --- | --- | --- | --- | --- | --- | --- | --- | --- | --- | --- | --- | --- | --- | --- | --- | --- |
|  | GU→GC | AC→GC | GA→GC | UC→GC | GG→GC | CC→GC | NN→GC^a^ | F(GC)^a^ | GU→AU | AC→AU | AG→AU | CU→AU | AA→AU | UU→AU | NN→AU^a^ | F(AU)^a^ |
| *Sagittaria* | 0.6608 | 4.8084 | 1.0840 | 2.1300 | 1.0840 | 2.1300 | 11.8972 | 49.64 | 0.3157 | 3.4923 | 1.5470 | 0.5179 | 1.5470 | 0.5179 | 7.9378 | 17.22 |
| *Allium* | 0.4264 | 1.4841 | 1.0033 | 0.8556 | 1.0033 | 0.8556 | 5.6283 | 33.22 | 0.5781 | 1.2200 | 0.7033 | 1.3602 | 0.7033 | 1.3602 | 5.9251 | 37.02 |
| *Crocus* | 4.7017 | 12.7199 | 11.8038 | 2.0715 | 11.8038 | 2.0715 | 45.1722 | 67.14 | 1.6171 | 14.2825 | 2.3260 | 4.0597 | 2.3260 | 4.0597 | 28.6710 | 25.92 |
| *Aegilops* | 0.9951 | 10.4116 | 18.9168 | 25.9082 | 18.9168 | 25.9082 | 101.0567 | 73.44 | 0.3576 | 5.2061 | 12.9550 | 6.7980 | 12.9550 | 6.7980 | 45.0697 | 13.20 |
| *Agrostis* | 0.6287 | 2.8465 | 2.8465 | 2.8465 | 2.8465 | 2.8465 | 14.8612 | 59.80 | 0.1689 | 0.7649 | 0.7649 | 0.7649 | 0.7649 | 0.7649 | 3.9934 | 17.53 |
| *Eleusine* | 0.5828 | 3.5938 | 5.3336 | 8.0530 | 5.3336 | 8.0530 | 30.9498 | 51.06 | 0.6267 | 2.3512 | 5.2688 | 5.7359 | 5.2688 | 5.7359 | 24.9873 | 35.92 |
| *Hordeum* | 1.0410 | 2.2357 | 1.4716 | 0.5554 | 1.4716 | 0.5554 | 7.3307 | 51.72 | 0.5306 | 1.9663 | 0.4885 | 0.7501 | 0.4885 | 0.7501 | 4.9741 | 23.18 |
| *Oryza* | 0.5521 | 22.2025 | 22.2025 | 22.2025 | 22.2025 | 22.2025 | 111.5646 | 82.22 | 0.0797 | 3.2039 | 3.2039 | 3.2039 | 3.2039 | 3.2039 | 16.0992 | 7.92 |
| *Pennisetum* | 1.2288 | 4.7485 | 2.9204 | 1.6169 | 2.9204 | 1.6169 | 15.0519 | 54.96 | 0.5066 | 4.8317 | 1.6453 | 1.2041 | 1.6453 | 1.2041 | 11.0371 | 23.06 |
| *Setaria* | 1.3161 | 2.7007 | 2.7007 | 2.7007 | 2.7007 | 2.7007 | 14.8196 | 63.53 | 0.7981 | 1.6377 | 1.6377 | 1.6377 | 1.6377 | 1.6377 | 8.9866 | 17.32 |
| *Sorghum* | 1.2821 | 15.7894 | 18.8816 | 13.5879 | 18.8816 | 13.5879 | 82.0105 | 68.58 | 0.9753 | 5.2306 | 4.5013 | 14.3642 | 4.5013 | 14.3642 | 43.9369 | 17.28 |
| *Musa* | 0.3142 | 2.1762 | 2.1762 | 2.1762 | 2.1762 | 2.1762 | 11.1952 | 45.64 | 0.0743 | 0.5147 | 0.5147 | 0.5147 | 0.5147 | 0.5147 | 2.6478 | 18.16 |
| *Curcuma* | 0.9098 | 2.5602 | 4.5157 | 1.6035 | 4.5157 | 1.6035 | 15.7084 | 46.24 | 0.5502 | 2.9566 | 1.8517 | 2.7309 | 1.8517 | 2.7309 | 12.6720 | 32.30 |
| *Eleutherococcus* | 0.9249 | 10.0628 | 10.0628 | 10.0628 | 10.0628 | 10.0628 | 51.2389 | 59.93 | 0.4234 | 4.6064 | 4.6064 | 4.6064 | 4.6064 | 4.6064 | 23.4554 | 20.84 |
| *Hedera* | 1.1407 | 7.1959 | 7.1959 | 7.1959 | 7.1959 | 7.1959 | 37.1202 | 55.59 | 0.5968 | 3.7645 | 3.7645 | 3.7645 | 3.7645 | 3.7645 | 19.4193 | 29.45 |
| *Schefflera* | 0.6328 | 3.0484 | 3.0484 | 3.0484 | 3.0484 | 3.0484 | 15.8748 | 55.54 | 0.2199 | 1.0593 | 1.0593 | 1.0593 | 1.0593 | 1.0593 | 5.5164 | 20.93 |
| *Carthamus* | 0.3928 | 5.0552 | 3.4544 | 7.3404 | 3.4544 | 7.3404 | 27.0376 | 43.96 | 0.3298 | 3.4102 | 4.9518 | 2.9008 | 4.9518 | 2.9008 | 19.4452 | 24.90 |
| *Helianthus* | 0.9311 | 6.6854 | 2.2185 | 3.4218 | 2.2185 | 3.4218 | 18.8971 | 48.20 | 0.3685 | 8.8677 | 4.5388 | 0.8780 | 4.5388 | 0.8780 | 20.0698 | 25.30 |
| *Lactuca* | 0.6047 | 4.2304 | 1.7453 | 2.5660 | 1.7453 | 2.5660 | 13.4577 | 35.88 | 0.4518 | 5.4482 | 3.3048 | 1.3039 | 3.3048 | 1.3039 | 15.1174 | 34.52 |
| *Gaillardia* | 0.1670 | 1.2625 | 1.2625 | 1.2625 | 1.2625 | 1.2625 | 6.4795 | 39.68 | 0.2309 | 1.7456 | 1.7456 | 1.7456 | 1.7456 | 1.7456 | 8.9589 | 26.86 |
| *Lasthenia* | 0.4759 | 1.3589 | 1.3589 | 1.3589 | 1.3589 | 1.3589 | 7.2704 | 43.32 | 0.6054 | 1.7289 | 1.7289 | 1.7289 | 1.7289 | 1.7289 | 9.2499 | 29.75 |
| *Sinosenecio* | 0.8230 | 1.9960 | 1.9960 | 1.9960 | 1.9960 | 1.9960 | 10.8030 | 46.94 | 0.6176 | 1.4980 | 1.4980 | 1.4980 | 1.4980 | 1.4980 | 8.1076 | 26.37 |
| *Arabidopsis* | 0.9208 | 2.0886 | 4.2897 | 0.8569 | 4.2897 | 0.8569 | 13.3026 | 49.00 | 0.5454 | 1.9646 | 0.8060 | 2.5410 | 0.8060 | 2.5410 | 9.2040 | 27.30 |
| *Noccaea* | 0.7246 | 3.0858 | 55.9427 | 5.4663 | 55.9427 | 5.4663 | 126.6284 | 37.34 | 0.3619 | 5.9334 | 10.5106 | 27.9372 | 10.5106 | 27.9372 | 83.1909 | 35.84 |
| *Cucumis* | 0.7834 | 6.3240 | 9.1003 | 7.7570 | 9.1003 | 7.7570 | 40.8220 | 62.44 | 0.8497 | 2.1568 | 2.6455 | 9.8711 | 2.6455 | 9.8711 | 28.0397 | 23.10 |
| *Rhododendron* | 1.2383 | 7.6088 | 5.2700 | 3.8813 | 5.2700 | 3.8813 | 27.1497 | 55.22 | 1.1037 | 4.6634 | 2.3789 | 4.6970 | 2.3789 | 4.6970 | 19.9189 | 30.16 |
| *Cyclamen* | 3.2545 | 2.9619 | 2.9619 | 2.9619 | 2.9619 | 2.9619 | 18.0640 | 74.72 | 0.7676 | 0.6985 | 0.6985 | 0.6985 | 0.6985 | 0.6985 | 4.2601 | 15.57 |
| *Arachis* | 0.4880 | 2.4238 | 2.9038 | 3.2310 | 2.9038 | 3.2310 | 15.1814 | 59.96 | 0.3463 | 1.0049 | 1.3395 | 2.0604 | 1.3395 | 2.0604 | 8.1510 | 17.64 |
| *Cicer* | 1.2080 | 7.1796 | 7.1751 | 4.6274 | 7.1751 | 4.6274 | 31.9926 | 38.87 | 0.4526 | 16.8004 | 10.8280 | 2.6886 | 10.8280 | 2.6886 | 44.2862 | 36.32 |
| *Cyamopsis* | 0.7049 | 2.3180 | 1.5261 | 1.1148 | 1.5261 | 1.1148 | 8.3047 | 40.30 | 0.4814 | 2.7235 | 1.3098 | 1.0423 | 1.3098 | 1.0423 | 7.9091 | 32.34 |
| *Glycine* | 0.8640 | 6.2883 | 1.3763 | 1.8394 | 1.3763 | 1.8394 | 13.5837 | 53.32 | 0.9091 | 2.8835 | 0.8435 | 1.4481 | 0.8435 | 1.4481 | 8.3758 | 25.72 |
| *Salvia* | 1.6247 | 1.6158 | 1.6158 | 1.6158 | 1.6158 | 1.6158 | 9.7037 | 62.36 | 0.3303 | 0.3285 | 0.3285 | 0.3285 | 0.3285 | 0.3285 | 1.9728 | 16.82 |
| *Antirrhinum* | 1.5540 | 1.3565 | 15.1379 | 1.5548 | 15.1379 | 1.5548 | 36.2959 | 60.40 | 0.1573 | 4.6670 | 5.3490 | 1.5319 | 5.3490 | 1.5319 | 18.5861 | 21.02 |
| *Persea* | 2.2338 | 15.0670 | 7.0958 | 3.5655 | 7.0958 | 3.5655 | 38.6234 | 80.82 | 0.7364 | 3.6419 | 0.8618 | 2.3391 | 0.8618 | 2.3391 | 10.7801 | 6.44 |
| *Euphorbia* | 1.7308 | 2.9297 | 2.9297 | 2.9297 | 2.9297 | 2.9297 | 16.3793 | 71.65 | 0.3921 | 0.6636 | 0.6636 | 0.6636 | 0.6636 | 0.6636 | 3.7101 | 10.93 |
| *Asarum* | 1.2612 | 2.6319 | 2.9712 | 0.4371 | 2.9712 | 0.4371 | 10.7097 | 50.56 | 0.3457 | 4.2742 | 0.7098 | 0.8144 | 0.7098 | 0.8144 | 7.6683 | 22.50 |
| *Eschscholzia* | 0.6939 | 7.2020 | 7.2020 | 7.2020 | 7.2020 | 7.2020 | 36.7039 | 75.70 | 0.0666 | 0.6912 | 0.6912 | 0.6912 | 0.6912 | 0.6912 | 3.5226 | 10.89 |
| *Adonis* | 0.9632 | 7.1140 | 20.0198 | 13.1465 | 20.0198 | 13.1465 | 74.4098 | 51.58 | 0.6258 | 6.9224 | 12.7925 | 13.0068 | 12.7925 | 13.0068 | 59.1468 | 32.60 |
| *Fragaria* | 1.5536 | 9.3928 | 7.6578 | 8.4508 | 7.6578 | 8.4508 | 43.1636 | 71.06 | 0.3971 | 7.2754 | 6.5457 | 1.9575 | 6.5457 | 1.9575 | 24.6789 | 14.08 |
| *Malus* | 0.6543 | 1.3756 | 1.2870 | 1.0141 | 1.2870 | 1.0141 | 6.6321 | 57.96 | 0.2780 | 0.7886 | 0.5813 | 0.5469 | 0.5813 | 0.5469 | 3.3230 | 14.12 |
| *Rosa* | 1.3107 | 2.9672 | 22.0268 | 1.0246 | 22.0268 | 1.0246 | 50.3807 | 48.70 | 0.2421 | 7.8167 | 2.6992 | 4.0692 | 2.6992 | 4.0692 | 21.5956 | 23.70 |
| *Cedrela* | 0.0578 | 12.1897 | 12.1897 | 12.1897 | 12.1897 | 12.1897 | 61.0063 | 71.36 | 0.0220 | 4.6319 | 4.6319 | 4.6319 | 4.6319 | 4.6319 | 23.1815 | 17.46 |
| *Sedum* | 1.0407 | 0.9858 | 0.9858 | 0.9858 | 0.9858 | 0.9858 | 5.9697 | 43.58 | 0.5468 | 0.5180 | 0.5180 | 0.5180 | 0.5180 | 0.5180 | 3.1368 | 25.77 |
| *Ribes* | 1.0666 | 2.9167 | 3.2462 | 1.3763 | 3.2462 | 1.3763 | 13.2283 | 42.46 | 0.5698 | 3.6966 | 1.7443 | 1.7343 | 1.7443 | 1.7343 | 11.2236 | 34.68 |
| *Paeonia* | 0.4073 | 4.9129 | 4.9129 | 4.9129 | 4.9129 | 4.9129 | 24.9718 | 55.98 | 0.4585 | 5.5304 | 5.5304 | 5.5304 | 5.5304 | 5.5304 | 28.1105 | 26.97 |
| *Petunia* | 0.6677 | 12.1309 | 12.1309 | 12.1309 | 12.1309 | 12.1309 | 61.3222 | 57.33 | 0.2426 | 4.4076 | 4.4076 | 4.4076 | 4.4076 | 4.4076 | 22.2806 | 20.25 |

^a^ NN→GC/AU represent the sum substitution rates from the six intermediates to GC or AU; F_(GC)_/ F_(AU)_ represent the GC or AU frequency in ITS2 rate matrix

Table S4. Mismatches transformation inferring from the best-fit double-substitution rate matrix at initial states

| matrix | Mismatches transformation | | | | | | | |
| --- | --- | --- | --- | --- | --- | --- | --- | --- |
|  | AG | | AC | | GU | | CU | |
|  | AG→AU | AG→CG | AC→AU | AC→GC | GU→AU | GU→GC | CU→AU | CU→CG |
| *Sagittaria* | 1.0815 | 1.2005 | 7.3481 | 12.4277 | 0.8034 | 2.1296 | 0.4529 | 1.8292 |
| *Allium* | 0.9187 | 0.7110 | 4.6730 | 4.9305 | 1.3083 | 1.4349 | 0.6483 | 0.9693 |
| *Crocus* | 3.0466 | 6.7431 | 13.3289 | 34.1807 | 1.2117 | 4.2495 | 1.9228 | 7.8127 |
| *Aegilops* | 3.2913 | 8.0661 | 8.6003 | 25.7120 | 0.5193 | 2.8828 | 1.4531 | 9.8397 |
| *Agrostis* | 0.0090 | 0.0536 | 0.0090 | 0.0536 | 0.2687 | 1.6087 | 0.0090 | 0.0536 |
| *Eleusine* | 3.7319 | 3.7887 | 6.0422 | 8.0179 | 1.1296 | 1.6480 | 2.5968 | 4.9522 |
| *Hordeum* | 0.8130 | 1.5871 | 7.9101 | 18.6598 | 1.0084 | 3.3532 | 0.4773 | 1.9179 |
| *Oryza* | 0.0211 | 0.2083 | 0.0211 | 0.2083 | 0.1521 | 1.5020 | 0.0211 | 0.2083 |
| *Pennisetum* | 0.8146 | 0.9995 | 10.3825 | 18.1692 | 1.0306 | 2.6834 | 0.3839 | 1.4255 |
| *Setaria* | 0.0247 | 0.0452 | 0.0247 | 0.0452 | 0.7893 | 1.4453 | 0.0247 | 0.0452 |
| *Sorghum* | 1.0506 | 2.1596 | 18.0348 | 45.9647 | 0.9566 | 3.8187 | 0.5410 | 2.6776 |
| *Musa* | 0.0187 | 0.0874 | 0.0187 | 0.0874 | 0.2944 | 1.3759 | 0.0187 | 0.0874 |
| *Curcuma* | 0.8798 | 0.8959 | 10.5611 | 15.4281 | 0.9835 | 1.8302 | 0.4814 | 1.2853 |
| *Eleutherococcus* | 0.0226 | 0.0568 | 0.0226 | 0.0568 | 0.9373 | 2.3612 | 0.0226 | 0.0568 |
| *Hedera* | 0.0619 | 0.1360 | 0.0619 | 0.1360 | 1.2119 | 2.6615 | 0.0619 | 0.1360 |
| *Schefflera* | 0.0370 | 0.1004 | 0.0370 | 0.1004 | 0.7731 | 2.0944 | 0.0370 | 0.1004 |
| *Carthamus* | 2.0666 | 2.2218 | 8.0361 | 13.1618 | 0.7108 | 1.6705 | 0.9453 | 3.3847 |
| *Helianthus* | 3.4100 | 3.7200 | 13.3357 | 21.3677 | 0.8631 | 1.9743 | 1.6263 | 5.4638 |
| *Lactuca* | 0.9182 | 1.0423 | 6.7913 | 10.8955 | 0.8242 | 1.8179 | 0.4726 | 1.4731 |
| *Gaillardia* | 0.0779 | 0.0687 | 0.0779 | 0.0687 | 0.9011 | 0.7949 | 0.0779 | 0.0687 |
| *Lasthenia* | 0.0535 | 0.0498 | 0.0535 | 0.0498 | 0.8904 | 0.8291 | 0.0535 | 0.0498 |
| *Sinosenecio* | 0.0379 | 0.0706 | 0.0379 | 0.0706 | 0.6647 | 1.2373 | 0.0379 | 0.0706 |
| *Arabidopsis* | 1.6184 | 2.3584 | 5.9226 | 10.3255 | 1.2050 | 2.4599 | 1.1552 | 2.8215 |
| *Noccaea* | 3.2411 | 3.6143 | 17.2294 | 25.5715 | 1.6131 | 2.8656 | 2.0346 | 4.8104 |
| *Cucumis* | 2.2223 | 5.4198 | 10.2519 | 26.9095 | 2.9174 | 8.4178 | 1.8783 | 5.8333 |
| *Rhododendron* | 1.1080 | 1.5807 | 19.6811 | 34.7261 | 1.6113 | 3.4418 | 0.7400 | 1.9550 |
| *Cyclamen* | 0.0265 | 0.0692 | 0.0265 | 0.0692 | 0.7324 | 1.9143 | 0.0265 | 0.0692 |
| *Arachis* | 2.1006 | 5.4185 | 5.9818 | 18.1141 | 0.7996 | 3.6856 | 1.1756 | 6.3611 |
| *Cicer* | 5.4834 | 4.6753 | 18.3991 | 22.8742 | 1.4473 | 2.0554 | 3.2921 | 6.8172 |
| *Cyamopsis* | 2.2877 | 1.8646 | 5.0621 | 5.9982 | 0.9966 | 1.3178 | 1.4102 | 2.7107 |
| *Glycine* | 1.1622 | 1.4103 | 8.0886 | 12.7787 | 1.3212 | 2.5163 | 0.7405 | 1.8360 |
| *Salvia* | 0.0195 | 0.1522 | 0.0195 | 0.1327 | 0.2758 | 1.8761 | 0.0195 | 0.1522 |
| *Antirrhinum* | 1.9637 | 3.3322 | 12.8699 | 26.4956 | 1.3149 | 3.6230 | 1.2094 | 4.0426 |
| *Persea* | 0.6134 | 2.3786 | 12.9312 | 56.9385 | 0.6123 | 4.9629 | 0.2935 | 2.7010 |
| *Euphorbia* | 0.0198 | 0.1026 | 0.0198 | 0.1026 | 0.3852 | 1.9925 | 0.0198 | 0.1026 |
| *Asarum* | 0.4146 | 0.4187 | 13.9840 | 19.8792 | 1.4501 | 2.5133 | 0.2416 | 0.5893 |
| *Eschscholzia* | 0.0182 | 0.1871 | 0.0182 | 0.1871 | 0.1417 | 1.4587 | 0.0182 | 0.1871 |
| *Adonis* | 4.1348 | 4.7176 | 16.7338 | 23.8544 | 2.5777 | 4.1417 | 2.9362 | 5.8943 |
| *Fragaria* | 4.7714 | 9.0543 | 11.1903 | 26.3527 | 1.0375 | 3.7348 | 2.5152 | 11.2364 |
| *Malus* | 1.5182 | 3.2118 | 2.5076 | 6.4202 | 0.3792 | 1.5112 | 0.8059 | 3.8871 |
| *Rosa* | 3.8152 | 5.4751 | 44.2080 | 81.5369 | 1.5889 | 3.8675 | 2.2494 | 7.0367 |
| *Cedrela* | 0.0732 | 0.3189 | 0.0732 | 0.2330 | 0.4237 | 1.3486 | 0.0732 | 0.3189 |
| *Sedum* | 0.0561 | 0.1342 | 0.0561 | 0.1342 | 0.5002 | 1.1962 | 0.0561 | 0.1342 |
| *Ribes* | 2.7160 | 3.6120 | 12.7812 | 21.9287 | 1.3420 | 2.9548 | 1.6405 | 4.6598 |
| *Paeonia* | 0.1355 | 0.1549 | 0.1355 | 0.1549 | 1.3192 | 1.5078 | 0.1355 | 0.1549 |
| *Petunia* | 0.1782 | 0.4192 | 0.1782 | 0.4192 | 0.6414 | 1.5085 | 0.1782 | 0.4192 |
| *Acorus* | 2.1035 | 6.7501 | 3.2721 | 12.3146 | 0.2711 | 2.0356 | 0.8988 | 7.9165 |
| *Asparagus* | 0.0180 | 0.1113 | 0.0180 | 0.1113 | 0.3226 | 1.9891 | 0.0180 | 0.1113 |
| *Iris* | 0.0904 | 0.5412 | 0.4812 | 3.1585 | 0.5276 | 2.8809 | 0.0904 | 0.5412 |
| *Phalaenopsis* | 0.5846 | 1.2803 | 5.6867 | 15.5669 | 0.5653 | 2.7253 | 0.2656 | 1.6004 |
| *Habenaria* | 2.2156 | 1.6276 | 15.4986 | 16.2127 | 1.8763 | 1.9724 | 1.5483 | 2.3177 |
| *Holcoglossum* | 2.9709 | 7.3803 | 11.8838 | 33.5421 | 1.1414 | 4.5105 | 1.8675 | 8.3855 |
| *Smilax* | 0.0239 | 0.7519 | 0.0239 | 0.7519 | 0.2216 | 6.9799 | 0.0239 | 0.7519 |
| *Avena* | 0.0097 | 0.2100 | 0.0097 | 0.2100 | 0.1467 | 3.1780 | 0.0097 | 0.2100 |
| *Cenchrus* | 0.0599 | 0.0664 | 0.0599 | 0.0664 | 0.9961 | 1.1042 | 0.0599 | 0.0664 |
| *Cynodon* | 7.9084 | 8.7107 | 20.4901 | 29.5966 | 2.3925 | 4.0109 | 5.1959 | 11.4233 |
| *Eragrostis* | 1.2894 | 2.0134 | 6.5181 | 12.6638 | 1.5358 | 3.7135 | 0.8327 | 2.5051 |
| *Panicum* | 0.6837 | 1.0494 | 6.1868 | 12.0909 | 1.2890 | 3.4172 | 0.3959 | 1.3362 |
| *Leymus* | 0.0272 | 0.1410 | 0.0272 | 0.1410 | 0.4538 | 2.3535 | 0.0272 | 0.1410 |
| *Lolium* | 0.0278 | 0.1059 | 0.0278 | 0.1059 | 0.4839 | 1.8414 | 0.0278 | 0.1059 |
| *Phyllostachys* | 0.0975 | 0.4212 | 0.0975 | 0.4212 | 0.8608 | 3.7203 | 0.0975 | 0.4212 |
| *Puccinellia* | 0.1010 | 0.2685 | 0.1010 | 0.2685 | 1.0595 | 2.8167 | 0.1010 | 0.2685 |
| *Triticum* | 0.0203 | 0.1026 | 0.0203 | 0.1026 | 0.4947 | 2.4997 | 0.0203 | 0.1026 |
| *Zingiber* | 0.0269 | 0.1079 | 0.0269 | 0.1079 | 0.5663 | 2.2696 | 0.0269 | 0.1079 |
| *Apium* | 0.0761 | 0.0996 | 0.0761 | 0.0996 | 0.9837 | 1.2870 | 0.0761 | 0.0996 |
| *Angelica* | 1.7031 | 3.0562 | 5.3846 | 11.2428 | 1.3294 | 3.3584 | 1.2098 | 3.5561 |
| *Bupleurum* | 0.0572 | 0.1337 | 0.0572 | 0.1337 | 0.6815 | 1.5920 | 0.0572 | 0.1337 |
| *Aralia* | 1.4389 | 3.3471 | 6.0464 | 15.4109 | 1.1720 | 3.6342 | 1.0794 | 3.6673 |
| *Dendropanax* | 0.6493 | 1.3970 | 3.8995 | 9.4796 | 0.8967 | 2.6160 | 0.4789 | 1.5784 |
| *Panax* | 0.0662 | 0.1887 | 0.0662 | 0.1887 | 0.6925 | 1.9735 | 0.0662 | 0.1887 |
| *Macropanax* | 0.0892 | 0.2419 | 0.0892 | 0.2419 | 0.7480 | 2.0278 | 0.0892 | 0.2419 |
| *Oplopanax* | 0.1814 | 0.3544 | 0.1814 | 0.3544 | 0.6124 | 1.1960 | 0.1814 | 0.3544 |
| *Barnadesia* | 1.8571 | 2.5897 | 9.3367 | 15.9530 | 1.7783 | 3.6574 | 1.2592 | 3.1731 |
| *Cynara* | 0.0930 | 0.2007 | 0.0930 | 0.2007 | 0.4482 | 0.9679 | 0.0930 | 0.2007 |
| *Stevia* | 1.0433 | 0.8721 | 6.6406 | 8.6587 | 0.7143 | 1.1727 | 0.5312 | 1.3604 |
| *Chaetanthera* | 0.4676 | 0.8469 | 2.6642 | 6.3895 | 0.3816 | 1.6067 | 0.2011 | 1.1215 |
| *Chrysanthemum* | 0.9157 | 1.2140 | 15.2419 | 25.7730 | 1.3195 | 2.7386 | 0.5849 | 1.5483 |
| *Dahlia* | 1.0586 | 1.3473 | 6.9014 | 11.8625 | 0.8586 | 2.0488 | 0.5646 | 1.8196 |
| *Flaveria* | 0.6615 | 0.5005 | 5.5728 | 6.6828 | 0.8578 | 1.2196 | 0.3520 | 0.7933 |
| *Lessingia* | 2.5010 | 2.1713 | 27.4582 | 33.2911 | 1.6435 | 2.1714 | 1.6435 | 3.0323 |
| *Melampodium* | 0.6214 | 0.7483 | 3.8225 | 6.1798 | 0.7478 | 1.6728 | 0.3345 | 1.0047 |
| *Senecio* | 0.0207 | 0.0293 | 0.0207 | 0.0293 | 0.6210 | 0.8806 | 0.0207 | 0.0293 |
| *Taraxacum* | 0.0332 | 0.0460 | 0.0332 | 0.0460 | 0.7797 | 1.0805 | 0.0332 | 0.0460 |
| *Zinnia* | 0.0515 | 0.0507 | 0.0515 | 0.0507 | 0.8023 | 0.7907 | 0.0515 | 0.0507 |
| *Brassica* | 0.0232 | 0.0636 | 0.0232 | 0.0636 | 0.4804 | 1.3186 | 0.0232 | 0.0636 |
| *Draba* | 3.3711 | 5.1125 | 11.7037 | 22.1663 | 1.0457 | 2.5115 | 2.1286 | 6.3847 |
| *Mesembryanthemum* | 0.0751 | 0.4367 | 0.0751 | 0.4367 | 0.2979 | 1.7319 | 0.0751 | 0.4367 |
| *Silene* | 0.0195 | 0.0714 | 0.0195 | 0.0714 | 0.5863 | 2.1497 | 0.0195 | 0.0714 |
| *Limonium* | 0.9884 | 0.6749 | 5.3668 | 6.1733 | 0.7794 | 1.0273 | 0.5120 | 1.1370 |
| *Persicaria* | 0.7624 | 2.0511 | 3.5280 | 10.5785 | 0.8905 | 3.3743 | 0.5413 | 2.2860 |
| *Tamarix* | 0.0431 | 0.1293 | 0.0431 | 0.1293 | 0.8448 | 2.5343 | 0.0431 | 0.1293 |
| *Euonymus* | 0.0617 | 0.2189 | 0.0617 | 0.2189 | 0.9100 | 3.2305 | 0.0617 | 0.2189 |
| *Parnassia* | 1.4094 | 1.4554 | 3.7035 | 4.9195 | 1.1547 | 1.7505 | 0.9601 | 1.8722 |
| *Hemsleya* | 0.4069 | 1.3545 | 0.4069 | 1.3545 | 1.2987 | 4.3235 | 0.4069 | 1.3545 |
| *Viburnum* | 0.0357 | 0.0842 | 0.0357 | 0.0842 | 0.5383 | 1.2685 | 0.0357 | 0.0842 |
| *Actinidia* | 0.9858 | 1.0880 | 12.3968 | 17.4396 | 1.8063 | 2.8115 | 0.6990 | 1.3868 |
| *Diospyros* | 0.6957 | 3.3530 | 3.5719 | 19.2183 | 0.4282 | 4.1441 | 0.3465 | 3.7430 |
| *Vaccinium* | 0.4510 | 0.9130 | 8.5181 | 20.6926 | 1.0656 | 3.6146 | 0.2692 | 1.0955 |
| *Enkianthus* | 0.0573 | 0.0924 | 0.0573 | 0.0924 | 1.0148 | 1.6350 | 0.0573 | 0.0924 |
| *Lysimachia* | 0.0173 | 0.0574 | 0.0173 | 0.0574 | 0.4859 | 1.6131 | 0.0173 | 0.0574 |
| *Lathyrus* | 2.8297 | 2.3929 | 7.2791 | 8.8738 | 1.1164 | 1.5225 | 1.7547 | 3.4496 |
| *Lotus* | 0.9886 | 1.0069 | 5.1424 | 7.3603 | 1.1471 | 1.9127 | 0.6039 | 1.4150 |
| *Lupinus* | 0.1174 | 0.5242 | 0.1174 | 0.5242 | 0.5363 | 2.3948 | 0.1174 | 0.5242 |
| *Medicago* | 2.9604 | 2.7824 | 13.9060 | 17.3580 | 1.9088 | 2.6616 | 1.9955 | 3.6953 |
| *Trifolium* | 1.6644 | 1.3910 | 8.4510 | 10.3510 | 1.2425 | 1.7748 | 0.9738 | 2.0386 |
| *Vicia* | 1.8165 | 1.4746 | 10.8412 | 13.7777 | 1.0092 | 1.4656 | 1.0153 | 2.3086 |
| *Phaseolus* | 0.8595 | 1.2085 | 12.9359 | 21.7990 | 2.2049 | 4.3515 | 0.6124 | 1.4483 |
| *Vigna* | 1.2869 | 1.2685 | 11.7895 | 14.9333 | 2.2796 | 3.0841 | 0.9376 | 1.6300 |
| *Astragalus* | 1.0825 | 1.2168 | 6.9981 | 10.1472 | 1.2929 | 2.1619 | 0.7277 | 1.5696 |
| *Betula* | 0.0321 | 0.1116 | 0.0321 | 0.1116 | 0.6115 | 2.1239 | 0.0321 | 0.1116 |
| *Quercus* | 0.7199 | 1.3750 | 9.0852 | 20.6554 | 0.9754 | 3.0257 | 0.4433 | 1.6367 |
| *Juglans* | 0.2354 | 0.5731 | 0.2354 | 0.5731 | 1.1558 | 2.8140 | 0.2354 | 0.5731 |
| *Swertia* | 0.7496 | 1.3498 | 4.2175 | 9.1315 | 0.8741 | 2.5578 | 0.4613 | 1.6230 |
| *Coffea* | 0.0868 | 0.2271 | 0.0868 | 0.2271 | 1.0862 | 2.8401 | 0.0868 | 0.2271 |
| *Hedyotis* | 0.9613 | 2.1438 | 6.1680 | 16.2954 | 1.0981 | 4.1489 | 0.5674 | 2.5396 |
| *Kadua* | 0.0288 | 0.1037 | 0.0288 | 0.1037 | 0.5865 | 2.1157 | 0.0288 | 0.1037 |
| *Leptodermis* | 2.7989 | 5.9506 | 28.1494 | 64.9807 | 3.1917 | 8.2996 | 2.2884 | 6.4611 |
| *Fraxinus* | 0.9999 | 2.0777 | 8.2741 | 21.3330 | 0.5637 | 2.4899 | 0.4703 | 2.5781 |
| *Olea* | 1.1129 | 2.1035 | 5.9653 | 13.4847 | 0.8934 | 2.5584 | 0.7345 | 2.5157 |
| *Ligustrum* | 2.3306 | 2.6667 | 16.1790 | 25.0503 | 1.5430 | 3.0273 | 1.3592 | 3.6085 |
| *Mimulus* | 1.1694 | 2.7624 | 5.7099 | 15.6758 | 0.7460 | 3.1029 | 0.6641 | 3.2105 |
| *Plantago* | 1.6449 | 2.3432 | 4.0844 | 7.0539 | 0.9807 | 2.2513 | 1.0207 | 2.8408 |
| *Machilus* | 0.8259 | 3.4951 | 0.8259 | 3.4951 | 0.6805 | 2.8798 | 0.8259 | 3.4951 |
| *Hypericum* | 0.9286 | 1.5926 | 2.4613 | 4.9838 | 0.5547 | 1.4963 | 0.5904 | 1.8803 |
| *Populus* | 0.0169 | 0.3798 | 1.0528 | 0.6082 | 0.1630 | 3.9271 | 0.1018 | 0.0630 |
| *Gossypium* | 0.0715 | 0.1636 | 0.0715 | 0.1636 | 0.7755 | 1.7740 | 0.0715 | 0.1636 |
| *Oenothera* | 6.4374 | 11.5402 | 14.2408 | 30.4281 | 1.2206 | 3.4096 | 4.1314 | 13.7547 |
| *Aristolochia* | 0.0333 | 0.1077 | 0.0333 | 0.1077 | 0.4820 | 1.5589 | 0.0333 | 0.1077 |
| *Dysosma* | 0.7186 | 0.8353 | 7.4803 | 10.2214 | 2.1276 | 3.2726 | 0.5431 | 0.9819 |
| *Papaver* | 1.5054 | 2.1518 | 9.3542 | 16.4132 | 1.5795 | 3.3373 | 1.0184 | 2.6413 |
| *Clematis* | 0.7504 | 2.3449 | 5.3043 | 17.1810 | 4.6101 | 16.5752 | 0.6522 | 2.4307 |
| *Ficus* | 0.0303 | 0.1318 | 0.0303 | 0.1318 | 0.4564 | 1.9837 | 0.0303 | 0.1318 |
| *Prunus* | 1.2346 | 2.3918 | 8.5750 | 20.4287 | 0.7752 | 2.7031 | 0.6859 | 2.9412 |
| *Citrus* | 0.4998 | 1.8505 | 0.9088 | 2.8536 | 0.7707 | 3.3649 | 0.4998 | 1.8505 |
| *Paullinia* | 0.7341 | 2.0086 | 5.3293 | 17.3119 | 0.5987 | 3.1101 | 0.3867 | 2.3848 |
| *Cuscuta* | 0.5989 | 0.6368 | 1.8195 | 2.8862 | 0.4258 | 0.9545 | 0.2841 | 0.9501 |
| *Ipomoea* | 0.0279 | 0.0869 | 0.0279 | 0.0869 | 0.4374 | 1.3616 | 0.0279 | 0.0869 |
| *Capsicum* | 0.0630 | 0.2380 | 0.0630 | 0.2380 | 0.4905 | 1.8543 | 0.0630 | 0.2380 |
| *Solanum* | 0.3896 | 1.2589 | 1.9538 | 7.0844 | 1.1764 | 6.3138 | 0.2346 | 1.4125 |
| *Nicotiana* | 0.0586 | 0.1039 | 0.0586 | 0.1039 | 1.0349 | 1.8351 | 0.0586 | 0.1039 |
| *Lycium* | 0.0249 | 0.1357 | 0.0249 | 0.1357 | 0.4002 | 2.1810 | 0.0249 | 0.1357 |
| *Physalis* | 0.0403 | 0.1711 | 0.0403 | 0.1711 | 0.4384 | 1.8614 | 0.0403 | 0.1711 |

Table S5. Mismatches transformation inferring from the best-fit double-substitution rate matrix at equilibrium states

| Matrix | Mismatches transformation | | | | | | | |
| --- | --- | --- | --- | --- | --- | --- | --- | --- |
|  | AG | | AC | | GU | | CU | |
|  | AG→AU | AG→CG | AC→AU | AC→GC | GU→AU | GU→GC | CU→AU | CU→CG |
| *Sagittaria* | 1.5470 | 1.0840 | 3.4923 | 4.8084 | 0.3157 | 0.6608 | 0.5179 | 2.1300 |
| *Allium* | 0.7033 | 1.0033 | 1.2200 | 1.4841 | 0.5781 | 0.4264 | 1.3602 | 0.8556 |
| *Crocus* | 2.3260 | 11.8038 | 14.2825 | 12.7199 | 1.6171 | 4.7017 | 4.0597 | 2.0715 |
| *Aegilops* | 12.9550 | 18.9168 | 5.2061 | 10.4116 | 0.3576 | 0.9951 | 6.7980 | 25.9082 |
| *Agrostis* | 0.7649 | 2.8465 | 0.7649 | 2.8465 | 0.1689 | 0.6287 | 0.7649 | 2.8465 |
| *Eleusine* | 5.2688 | 5.3336 | 2.3512 | 3.5938 | 0.6267 | 0.5828 | 5.7359 | 8.0530 |
| *Hordeum* | 0.4885 | 1.4716 | 1.9663 | 2.2357 | 0.5306 | 1.0410 | 0.7501 | 0.5554 |
| *Oryza* | 3.2039 | 22.2025 | 3.2039 | 22.2025 | 0.0797 | 0.5521 | 3.2039 | 22.2025 |
| *Pennisetum* | 1.6453 | 2.9204 | 4.8317 | 4.7485 | 0.5066 | 1.2288 | 1.2041 | 1.6169 |
| *Setaria* | 1.6377 | 2.7007 | 1.6377 | 2.7007 | 0.7981 | 1.3161 | 1.6377 | 2.7007 |
| *Sorghum* | 4.5013 | 18.8816 | 5.2306 | 15.7894 | 0.9753 | 1.2821 | 14.3642 | 13.5879 |
| *Musa* | 0.5147 | 2.1762 | 0.5147 | 2.1762 | 0.0743 | 0.3142 | 0.5147 | 2.1762 |
| *Curcuma* | 1.8517 | 4.5157 | 2.9566 | 2.5602 | 0.5502 | 0.9098 | 2.7309 | 1.6035 |
| *Eleutherococcus* | 4.6064 | 10.0628 | 4.6064 | 10.0628 | 0.4234 | 0.9249 | 4.6064 | 10.0628 |
| *Hedera* | 3.7645 | 7.1959 | 3.7645 | 7.1959 | 0.5968 | 1.1407 | 3.7645 | 7.1959 |
| *Schefflera* | 1.0593 | 3.0484 | 1.0593 | 3.0484 | 0.2199 | 0.6328 | 1.0593 | 3.0484 |
| *Carthamus* | 4.9518 | 3.4544 | 3.4102 | 5.0552 | 0.3298 | 0.3928 | 2.9008 | 7.3404 |
| *Helianthus* | 4.5388 | 2.2185 | 8.8677 | 6.6854 | 0.3685 | 0.9311 | 0.8780 | 3.4218 |
| *Lactuca* | 3.3048 | 1.7453 | 5.4482 | 4.2304 | 0.4518 | 0.6047 | 1.3039 | 2.5660 |
| *Gaillardia* | 1.7456 | 1.2625 | 1.7456 | 1.2625 | 0.2309 | 0.1670 | 1.7456 | 1.2625 |
| *Lasthenia* | 1.7289 | 1.3589 | 1.7289 | 1.3589 | 0.6054 | 0.4759 | 1.7289 | 1.3589 |
| *Sinosenecio* | 1.4980 | 1.9960 | 1.4980 | 1.9960 | 0.6176 | 0.8230 | 1.4980 | 1.9960 |
| *Arabidopsis* | 0.8060 | 4.2897 | 1.9646 | 2.0886 | 0.5454 | 0.9208 | 2.5410 | 0.8569 |
| *Noccaea* | 10.5106 | 55.9427 | 5.9334 | 3.0858 | 0.3619 | 0.7246 | 27.9372 | 5.4663 |
| *Cucumis* | 2.6455 | 9.1003 | 2.1568 | 6.3240 | 0.8497 | 0.7834 | 9.8711 | 7.7570 |
| *Rhododendron* | 2.3789 | 5.2700 | 4.6634 | 7.6088 | 1.1037 | 1.2383 | 4.6970 | 3.8813 |
| *Cyclamen* | 0.6985 | 2.9619 | 0.6985 | 2.9619 | 0.7676 | 3.2545 | 0.6985 | 2.9619 |
| *Arachis* | 1.3395 | 2.9038 | 1.0049 | 2.4238 | 0.3463 | 0.4880 | 2.0604 | 3.2310 |
| *Cicer* | 10.8280 | 7.1751 | 16.8004 | 7.1796 | 0.4526 | 1.2080 | 2.6886 | 4.6274 |
| *Cyamopsis* | 1.3098 | 1.5261 | 2.7235 | 2.3180 | 0.4814 | 0.7049 | 1.0423 | 1.1148 |
| *Glycine* | 0.8435 | 1.3763 | 2.8835 | 6.2883 | 0.9091 | 0.8640 | 1.4481 | 1.8394 |
| *Salvia* | 0.3285 | 1.6158 | 0.3285 | 1.6158 | 0.3303 | 1.6247 | 0.3285 | 1.6158 |
| *Antirrhinum* | 5.3490 | 15.1379 | 4.6670 | 1.3565 | 0.1573 | 1.5540 | 1.5319 | 1.5548 |
| *Persea* | 0.8618 | 7.0958 | 3.6419 | 15.0670 | 0.7364 | 2.2338 | 2.3391 | 3.5655 |
| *Euphorbia* | 0.6636 | 2.9297 | 0.6636 | 2.9297 | 0.3921 | 1.7308 | 0.6636 | 2.9297 |
| *Asarum* | 0.7098 | 2.9712 | 4.2742 | 2.6319 | 0.3457 | 1.2612 | 0.8144 | 0.4371 |
| *Eschscholzia* | 0.6912 | 7.2020 | 0.6912 | 7.2020 | 0.0666 | 0.6939 | 0.6912 | 7.2020 |
| *Adonis* | 12.7925 | 20.0198 | 6.9224 | 7.1140 | 0.6258 | 0.9632 | 13.0068 | 13.1465 |
| *Fragaria* | 6.5457 | 7.6578 | 7.2754 | 9.3928 | 0.3971 | 1.5536 | 1.9575 | 8.4508 |
| *Malus* | 0.5813 | 1.2870 | 0.7886 | 1.3756 | 0.2780 | 0.6543 | 0.5469 | 1.0141 |
| *Rosa* | 2.6992 | 22.0268 | 7.8167 | 2.9672 | 0.2421 | 1.3107 | 4.0692 | 1.0246 |
| *Cedrela* | 4.6319 | 12.1897 | 4.6319 | 12.1897 | 0.0220 | 0.0578 | 4.6319 | 12.1897 |
| *Sedum* | 0.5180 | 0.9858 | 0.5180 | 0.9858 | 0.5468 | 1.0407 | 0.5180 | 0.9858 |
| *Ribes* | 1.7443 | 3.2462 | 3.6966 | 2.9167 | 0.5698 | 1.0666 | 1.7343 | 1.3763 |
| *Paeonia* | 5.5304 | 4.9129 | 5.5304 | 4.9129 | 0.4585 | 0.4073 | 5.5304 | 4.9129 |
| *Petunia* | 4.4076 | 12.1309 | 4.4076 | 12.1309 | 0.2426 | 0.6677 | 4.4076 | 12.1309 |
